# Supplementary material for: Objective comparison of methods to decode anomalous diffusion
Source: Nat Commun. 2021 Oct 29;12:6253. doi: 10.1038/s41467-021-26320-w (PMC8556353; doi:10.1038/s41467-021-26320-w)
Supplement: Supplementary file 1 — Supplementary Information [file 41467_2021_26320_MOESM1_ESM.pdf]

SUPPLEMENTARY INFORMATION

Objective comparison of methods to decode anomalous diffusion

Gorka Muñoz-Gil,[1] Giovanni Volpe,[2] Miguel Angel Garcia-March,[3] Erez Aghion,[4] Aykut Argun,[2] Chang Beom Hong,[5] Tom Bland,[6] Stefano Bo,[4] J. Alberto Conejero,[3] Nicolás Firbas,[3] Òscar Garibo i Orts,[3] Alessia Gentili,[7] Zihan Huang,[8] Jae-Hyung Jeon,[5] Hélène Kabbech,[9] Yeongjin Kim,[5] Patrycja Kowalek,[10] Diego Krapf,[11] Hanna Loch-Olszewska,[10] Michael A. Lomholt,[12] Jean-Baptiste Masson,[13] Philipp G. Meyer,[4] Seongyu Park,[5] Borja Requena,[1] Ihor Smal,[9] Taegeun Song,[5,14] Janusz Szwabiński,[10] Samudrajit Thapa,[15,16,17] Hippolyte Verdier,[18] Giorgio Volpe,[7] Artur Widera,[19] Maciej Lewenstein,[1,20] Ralf Metzler,[15] and Carlo Manzo[21,1,*]

[1] *ICFO – Institut de Ciències Fotòniques, The Barcelona Institute of Science and Technology, Av. Carl Friedrich Gauss 3, 08860 Castelldefels (Barcelona), Spain*
[2] *Department of Physics, University of Gothenburg, Origovägen 6B, SE-41296 Gothenburg, Sweden*
[3] *Instituto Universitario de Matemática Pura y Aplicada, Universitat Politècnica de València, Spain*
[4] *Max Planck Institute for the Physics of Complex Systems, Nöthnitzer Straße 38, DE-01187 Dresden, Germany*
[5] *Department of Physics, Pohang University of Science and Technology, Pohang 37673, Korea*
[6] *The Francis Crick Institute, 1 Midland Road, London, NW1 1AT, UK*
[7] *Department of Chemistry, University College London, 20 Gordon Street, London WC1H 0AJ, UK*
[8] *School of Physics and Electronics, Hunan University, Changsha 410082, China*
[9] *Department of Cell Biology, Erasmus MC, Rotterdam, the Netherlands*
[10] *Faculty of Pure and Applied Mathematics, Hugo Steinhaus Center, Wrocław University of Science and Technology, Wrocław, Poland*
[11] *Department of Electrical and Computer Engineering, Colorado State University, Fort Collins, Colorado 80523, USA*
[12] *PhyLife, Department of Physics, Chemistry and Pharmacy, University of Southern Denmark, DK-5230 Odense M, Denmark*
[13] *Institut Pasteur, Decision and Bayesian Computation lab, Paris*
[14] *Center for AI and Natural Sciences, Korea Institute for Advanced Study, Seoul, Korea*
[15] *Institute for Physics & Astronomy, University of Potsdam, Karl-Liebknecht-Str 24/25, D-14476 Potsdam-Golm, Germany*
[16] *Sackler Center for Computational Molecular and Materials Science, Tel Aviv University, Tel Aviv 69978, Israel*
[17] *School of Mechanical Engineering, Tel Aviv University, Tel Aviv 69978, Israel*
[18] *Institut Pasteur, Decision and Bayesian Computation lab, Paris, France*
[19] *Department of Physics and Research Center OPTIMAS, Technische Universität Kaiserslautern, 67663 Kaiserslautern, Germany*
[20] *ICREA, Pg. Lluís Companys 23, 08010 Barcelona, Spain*
[21] *Facultat de Ciències i Tecnologia, Universitat de Vic – Universitat Central de Catalunya (UVic-UCC), C. de la Laura,13, 08500 Vic, Spain*

(Dated: September 16, 2021)

* carlo.manzo@uvic.cat

SUPPLEMENTARY FIGURES AND TABLES

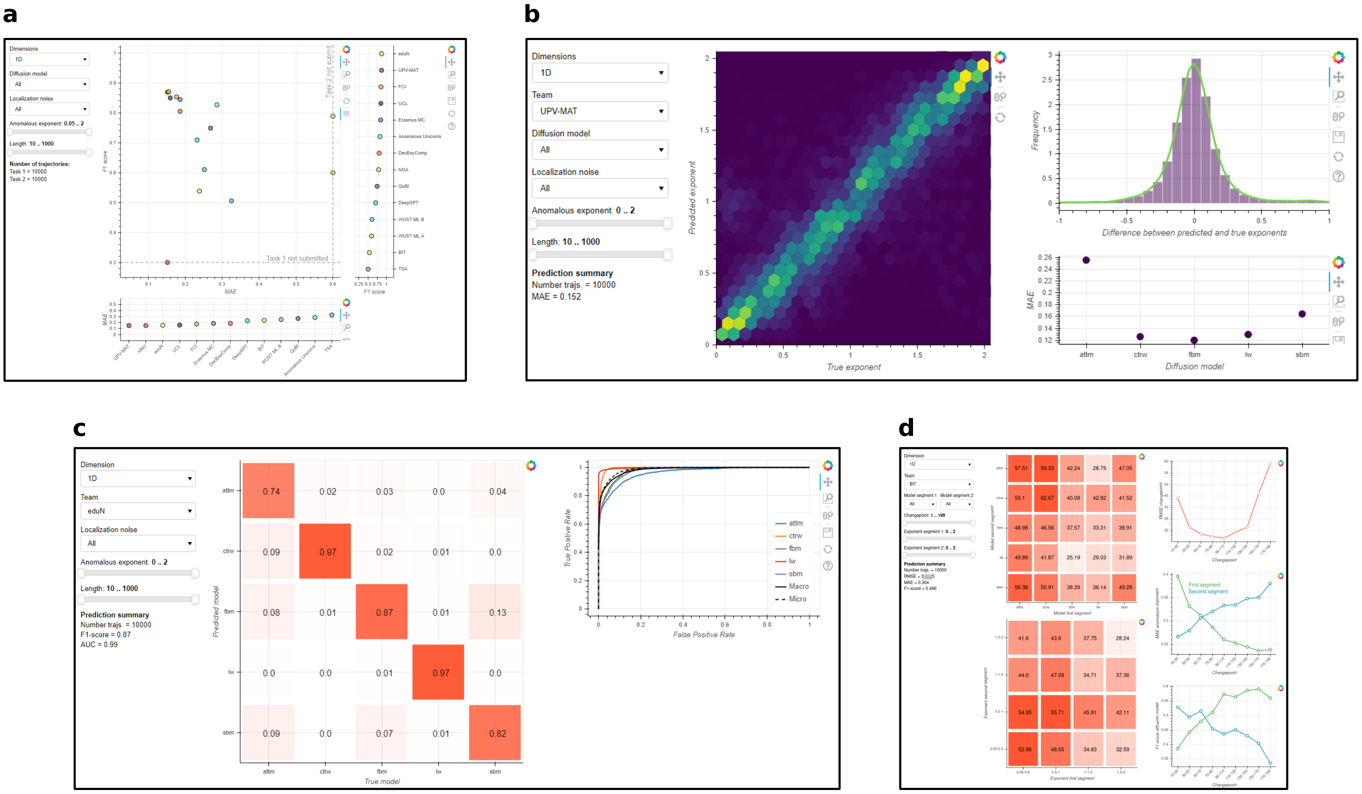

Supplementary Figure 1. **Screenshots of the interactive tool for performance comparison.** **a**, Summary of the results obtained for T1 and T2 according to corresponding challenge metrics. Hovering on each symbol reveals team name and scores. **b-d**, Plots of the metrics and estimators used to assess methods' performance for T1 (**b**), for T2 (**c**), and for T3 (**d**). For each task, plots can be displayed for user-selected subsets of the datasets. Sliders and buttons allow data selection based on task dimension, team, trajectory length, noise, α, diffusion model, or changepoint position. The interactive tool is available at http://andi-challenge.org/interactive-tool/.

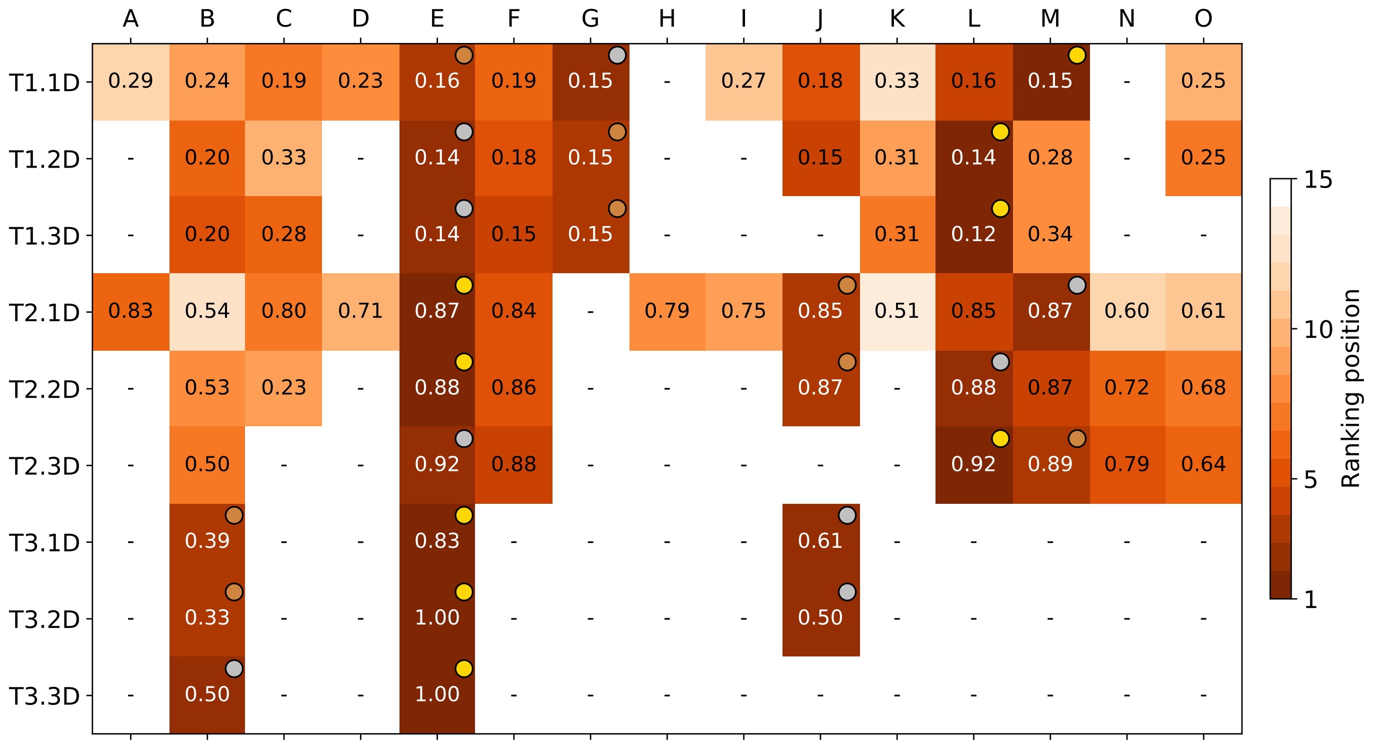

Supplementary Figure 2. **General ranking of the AnDi challenge.** Performance heatmap representing the value of the challenge metrics obtained by each team (A to O) for each task and dimension (T1.1D to T3.3D). The color code represents the relative position in the subtask leaderboard (the darker the color, the higher the rank). Top three teams of every subtask are labeled with a colored circle representing a medal (first – gold, second – silver, third – bronze).

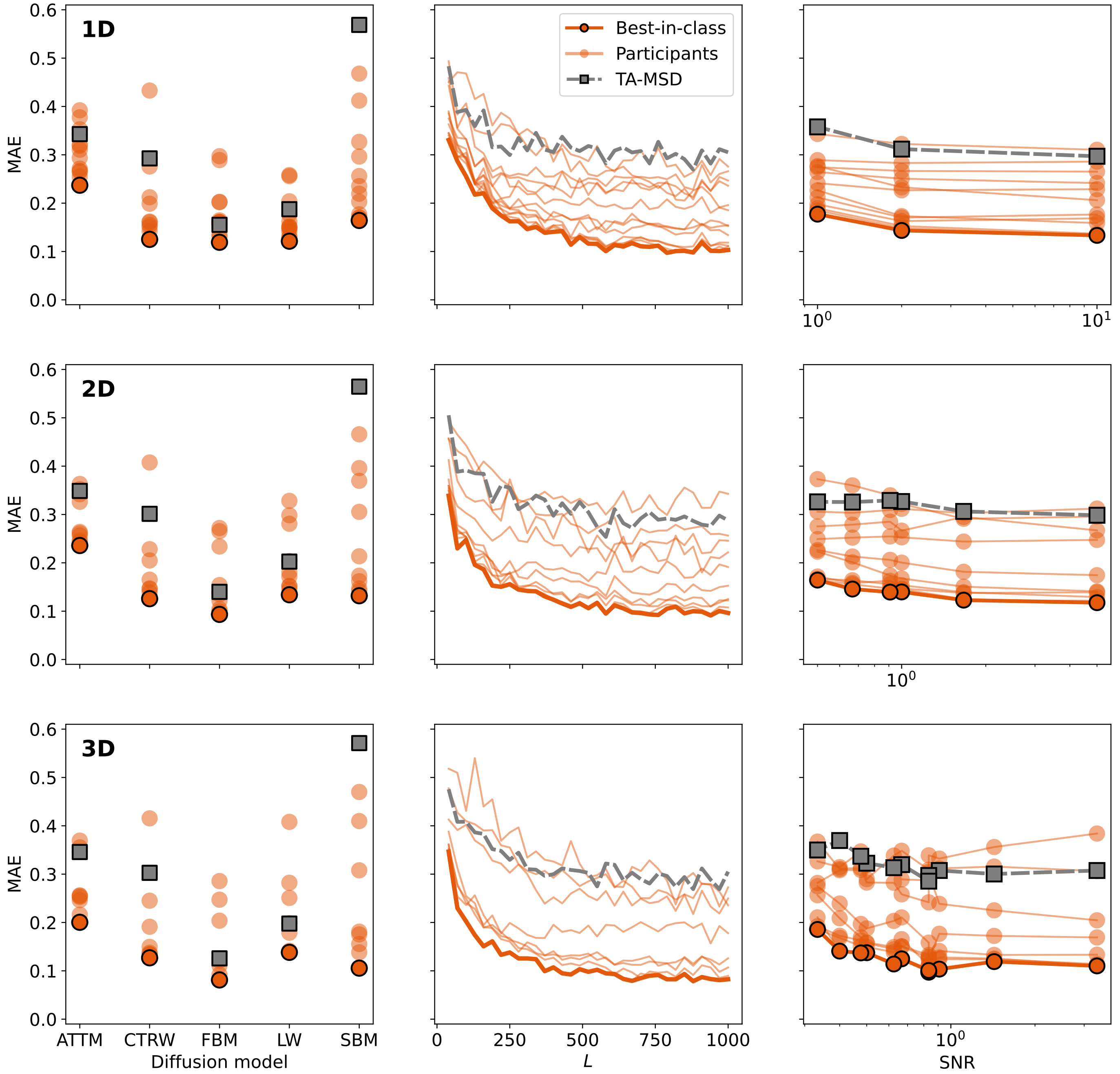

Supplementary Figure 3. **Comparison of method performance for T1.** MAE for all the submitted methods as a function of the diffusion model (left column), trajectory length (middle column), and SNR (right column). Rows show results obtained for different trajectory dimensions (from top to bottom, 1D, 2D, and 3D).

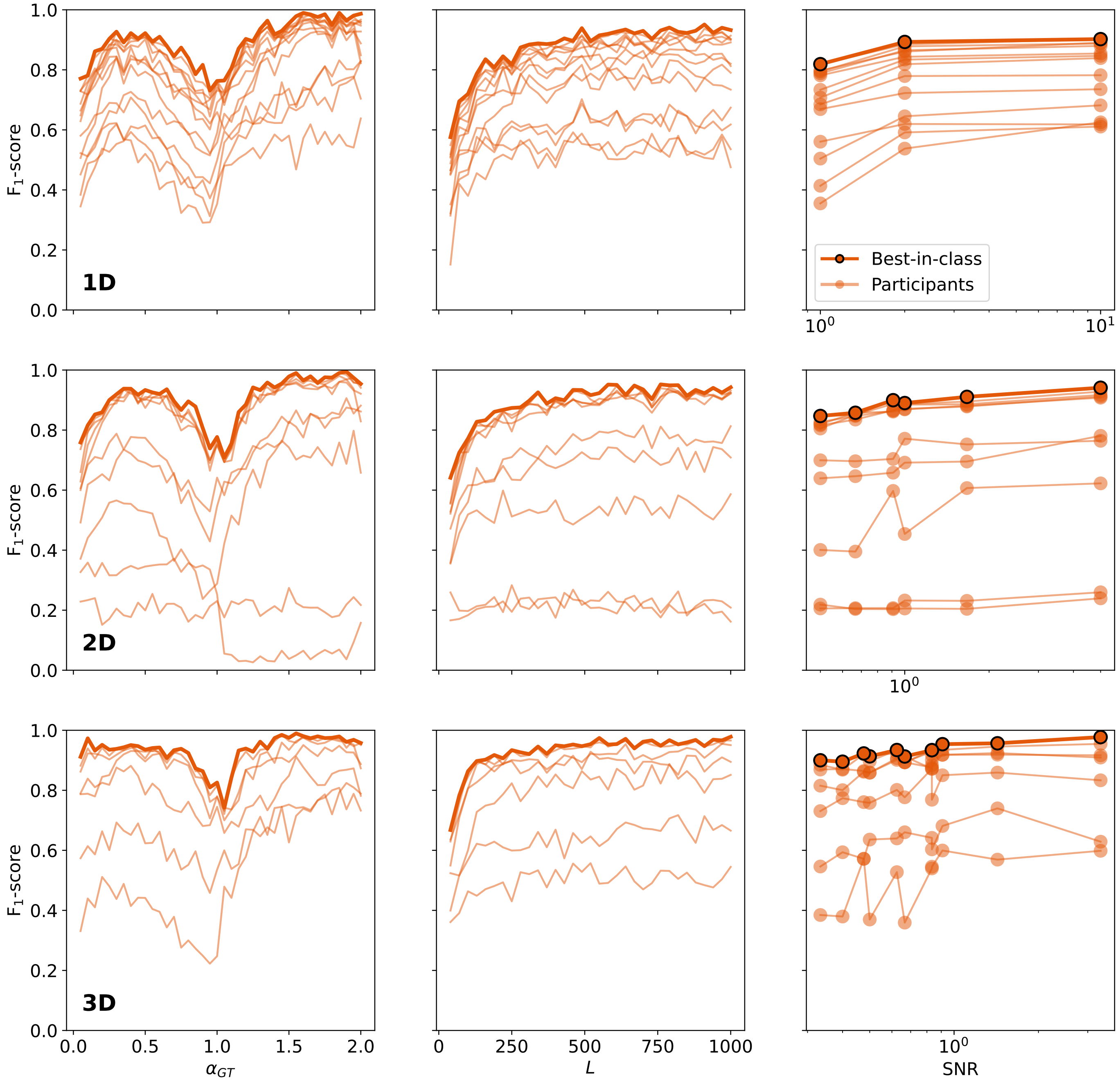

Supplementary Figure 4. **Comparison of method performance for T2.** F_1-score for all the submitted methods as a function of α_{GT} (left column), trajectory length (middle column), and SNR (right column). Rows show results obtained for different trajectory dimensions (from top to bottom, 1D, 2D, and 3D).

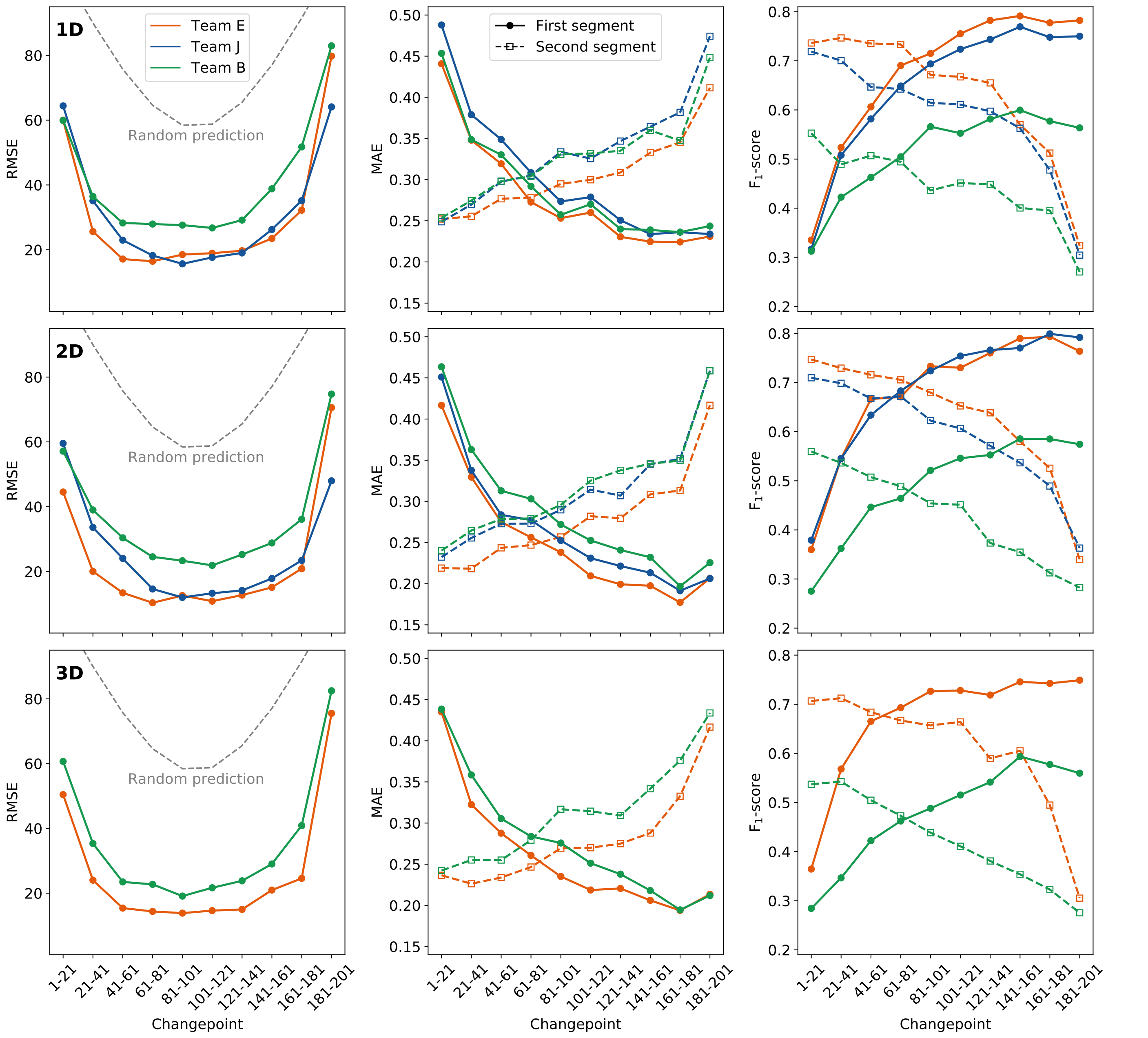

Supplementary Figure 5. **Comparison of method performance for T3.** RMSE for changepoint localization as a function of the changepoint position (left column), MAE for the prediction of α_{GT} of the first (solid) and second segment (dashed) as a function of the changepoint position (middle column), and F_1-score for classification of the diffusion model of the first (solid) and second segment (dashed) as a function of the changepoint position (right column). Rows show results obtained for different trajectory dimensions (from top to bottom, 1D, 2D, and 3D).

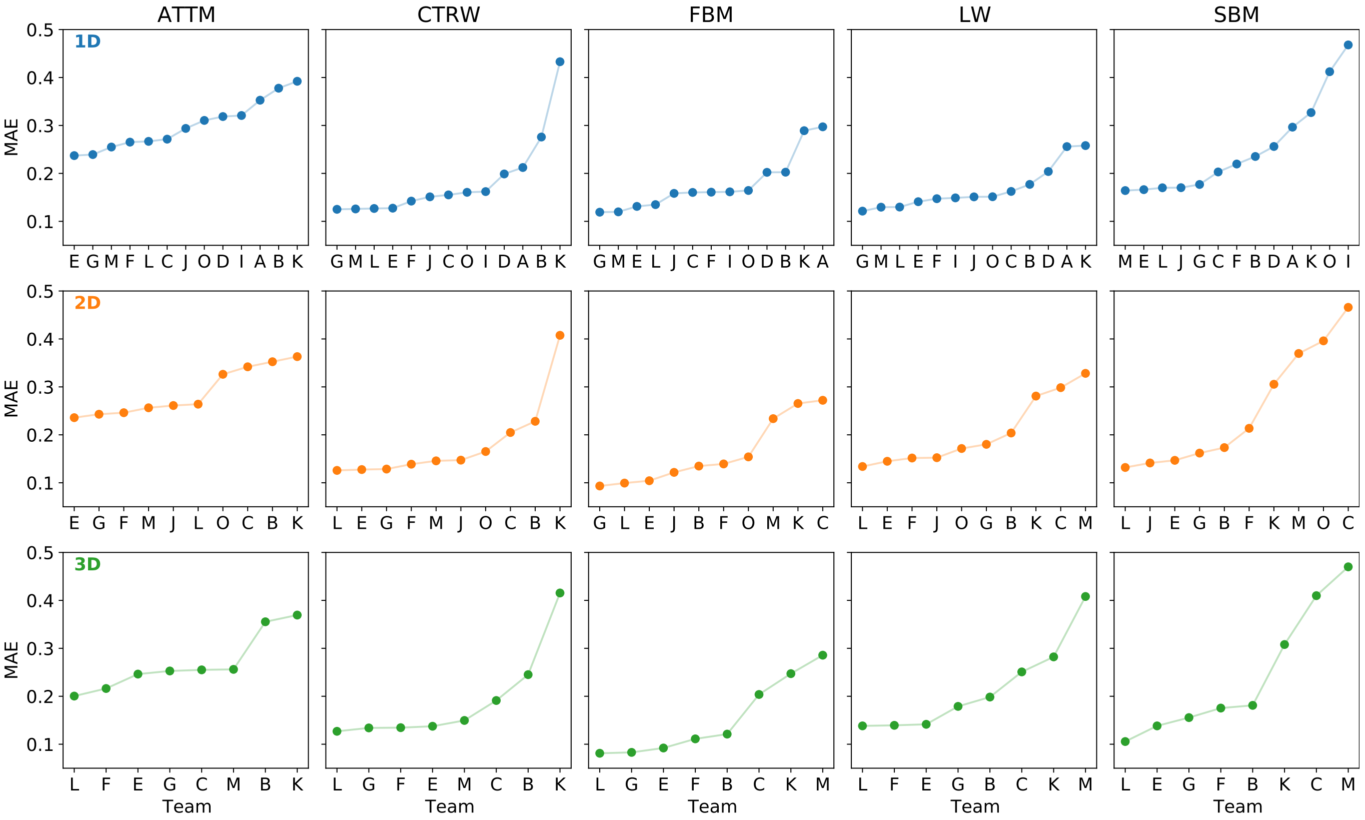

Supplementary Figure 6. **T1 leaderboard per diffusion model.** MAE for the prediction of α_{GT} obtained by submitted methods for each of the five diffusion model (columns). Rows show results obtained for different trajectory dimensions (from top to bottom, 1D, 2D, and 3D). Teams are ordered according to to their ranking in the leaderboard based on the MAE value.

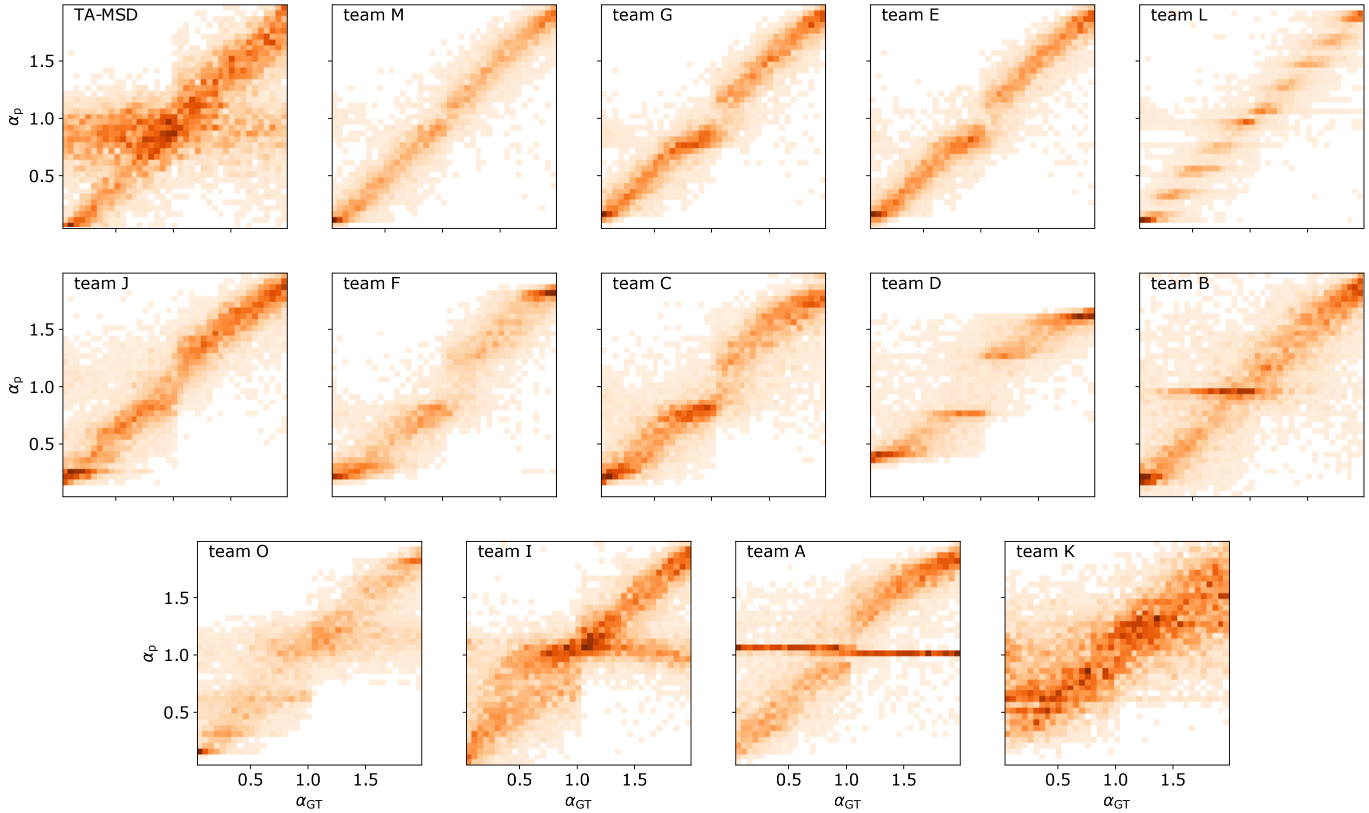

Supplementary Figure 7. **T1.1D methods' performance.** 2D histograms of the ground truth ($\alpha_{\rm GT}$) vs the predicted exponent ($\alpha_{\rm p}$) for all the submitted methods for T1.1D. Teams are ordered according to to their ranking in the leaderboard.

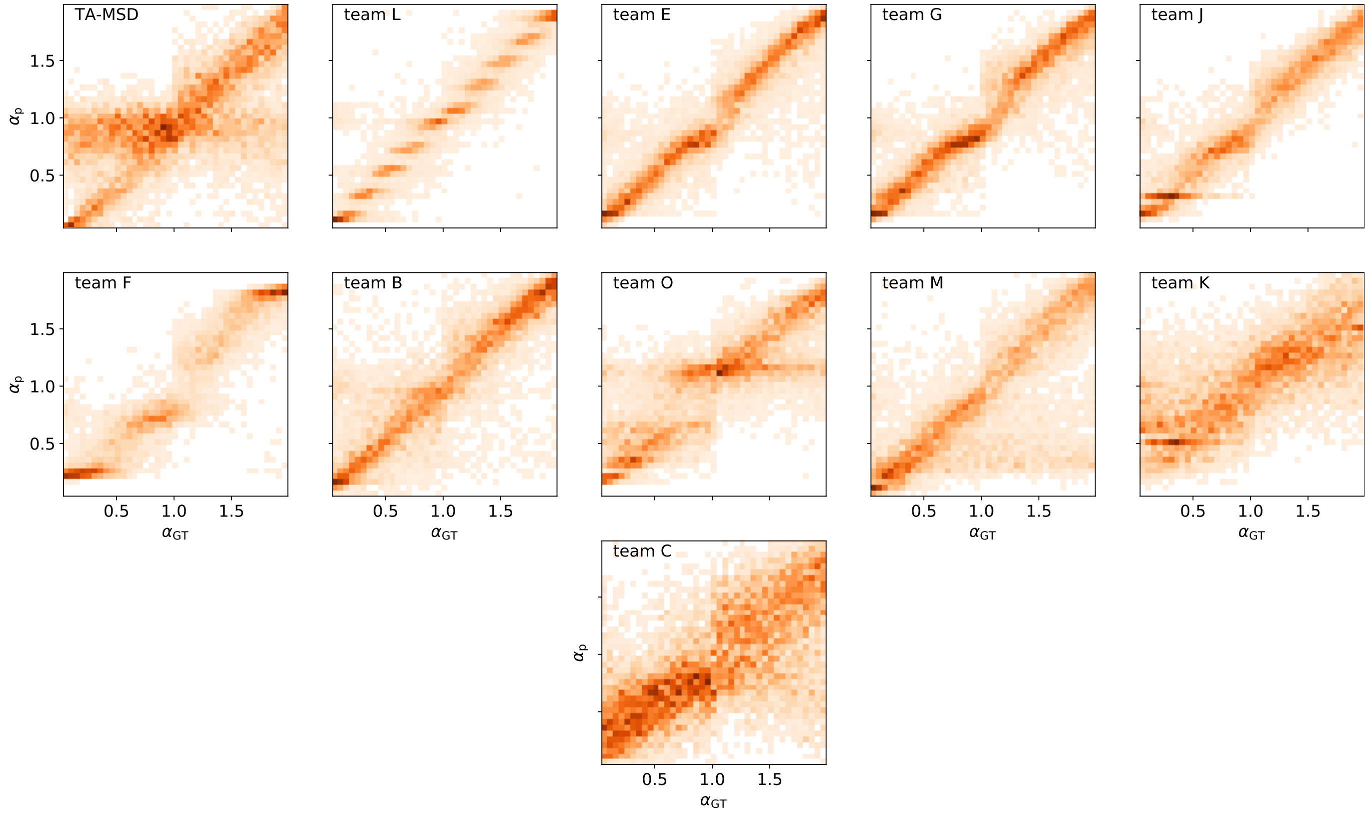

Supplementary Figure 8. **T1.2D methods' performance.** 2D histograms of the ground truth ($\alpha_{\rm GT}$) vs the predicted exponent ($\alpha_{\rm p}$) for all the submitted methods for T1.2D. Teams are ordered according to to their ranking in the leaderboard.

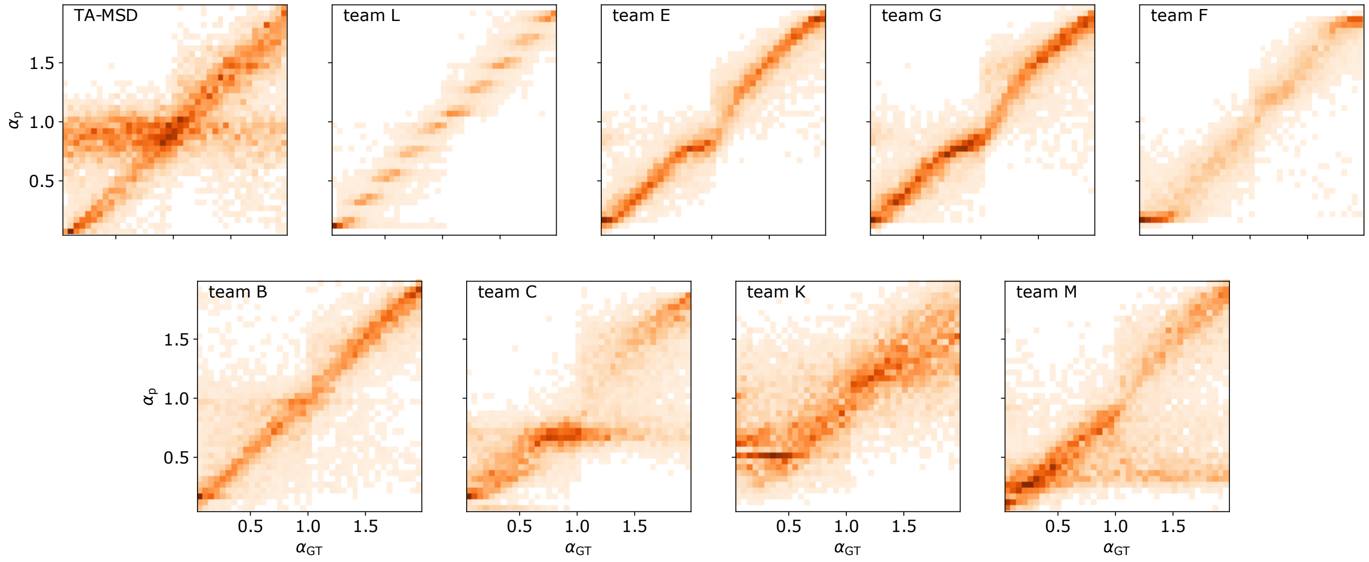

Supplementary Figure 9. **T1.3D methods' performance.** 2D histograms of the ground truth ($\alpha_{\rm GT}$) vs the predicted exponent ($\alpha_{\rm p}$) for all the submitted methods for T1.3D. Teams are ordered according to to their ranking in the leaderboard.

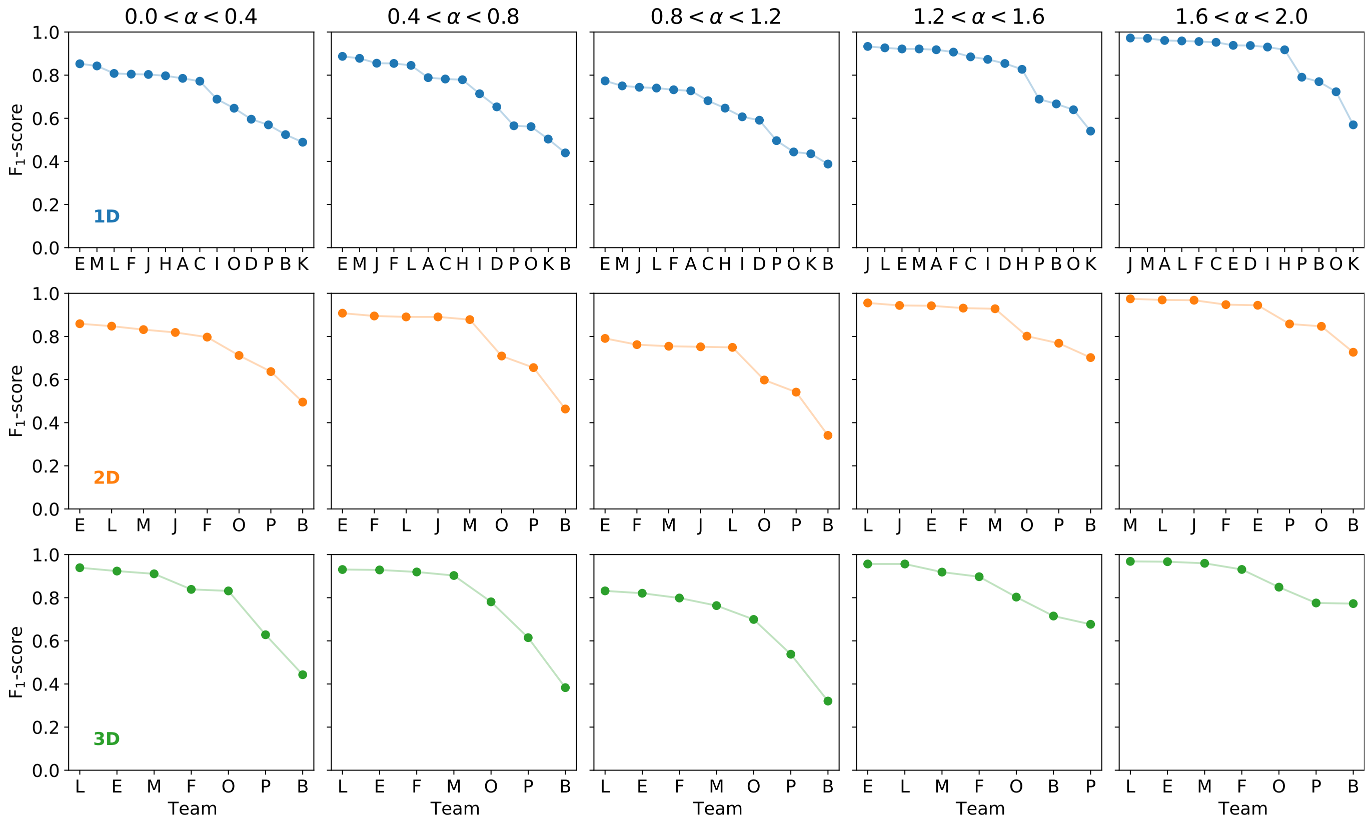

Supplementary Figure 10. **T2 leaderboard per range of α_{GT}.** F_1-score for the prediction of the diffusion model obtained by submitted methods for five ranges of α_{GT} (columns). Rows show results obtained for different trajectory dimensions (from top to bottom, 1D, 2D, and 3D). Teams are ordered according to to their ranking in the leaderboard based on the F_1-score value.

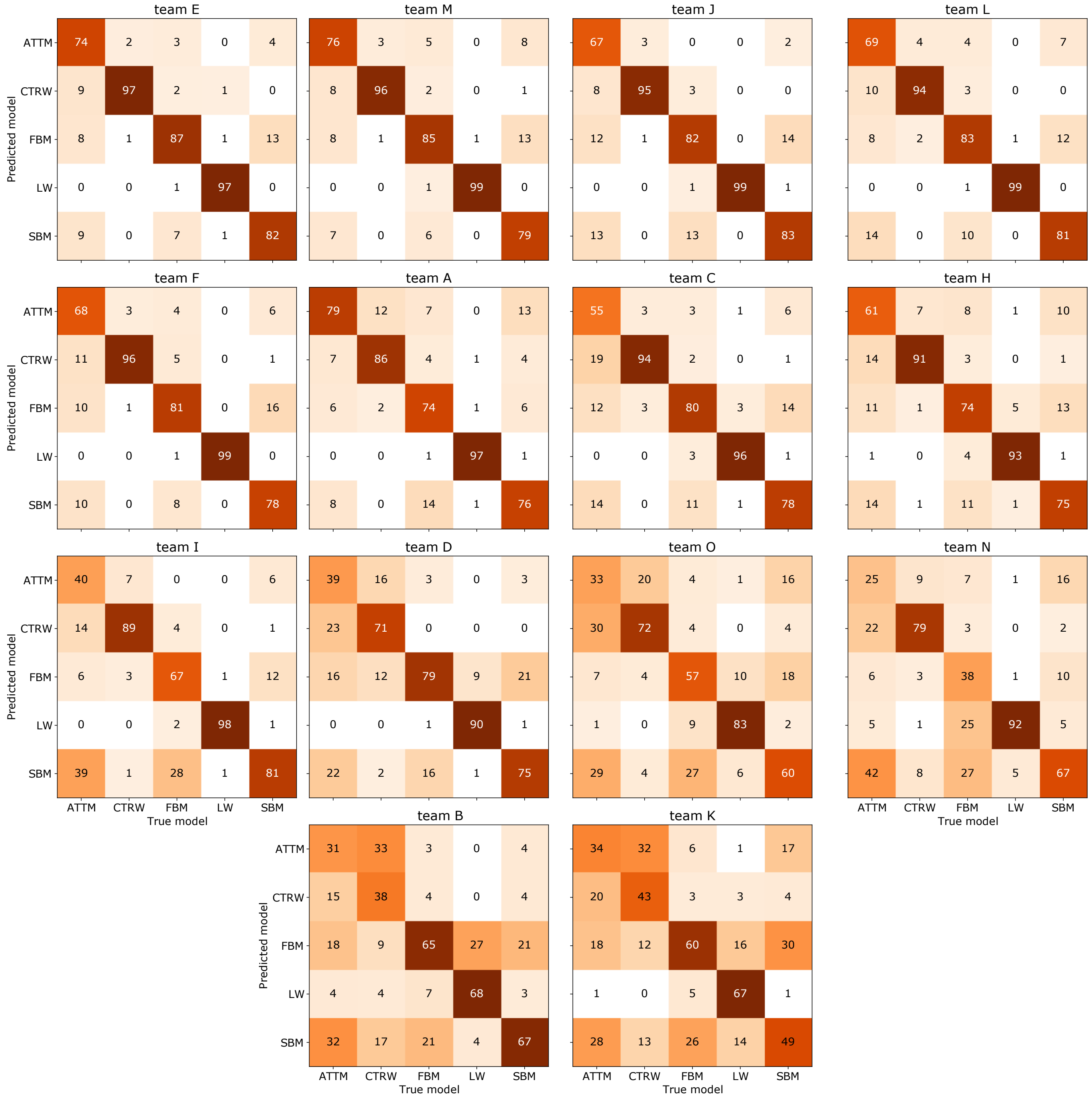

Supplementary Figure 11. **T2.1D methods' performance.** Confusion matrix of the ground truth model vs the predicted model for all the submitted methods for T2.1D. Teams are ordered according to to their ranking in the leaderboard. Numbers in matrix cells represent the number of correctly and incorrectly classified trajectories for each ground-truth model as percentages of the number of trajectories of the corresponding ground-truth model (column-based normalization). Thus, the percentages of correctly classified observations can be thought of as class-wise recalls.

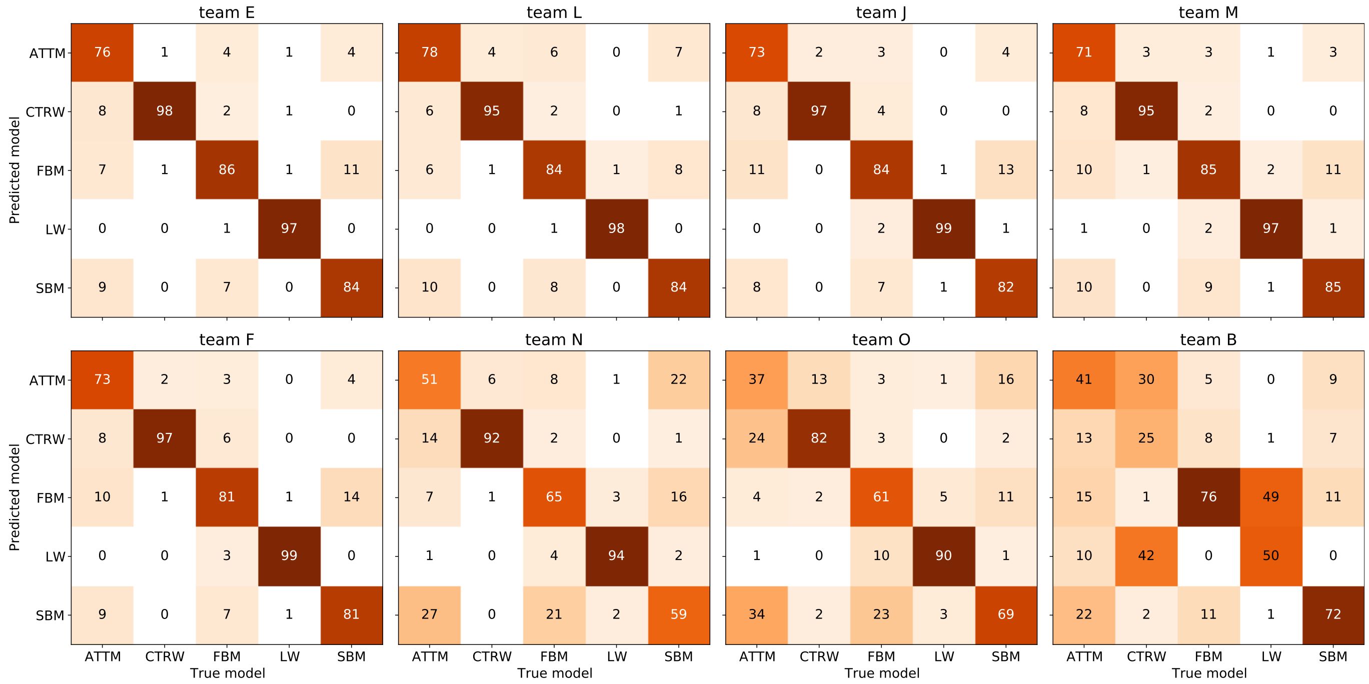

Supplementary Figure 12. **T2.2D methods' performance.** Confusion matrix of the ground truth model vs the predicted model for all the submitted methods for T2.2D. Teams are ordered according to to their ranking in the leaderboard. Numbers in matrix cells represent the number of correctly and incorrectly classified trajectories for each ground-truth model as percentages of the number of trajectories of the corresponding ground-truth model (column-based normalization). Thus, the percentages of correctly classified observations can be thought of as class-wise recalls.

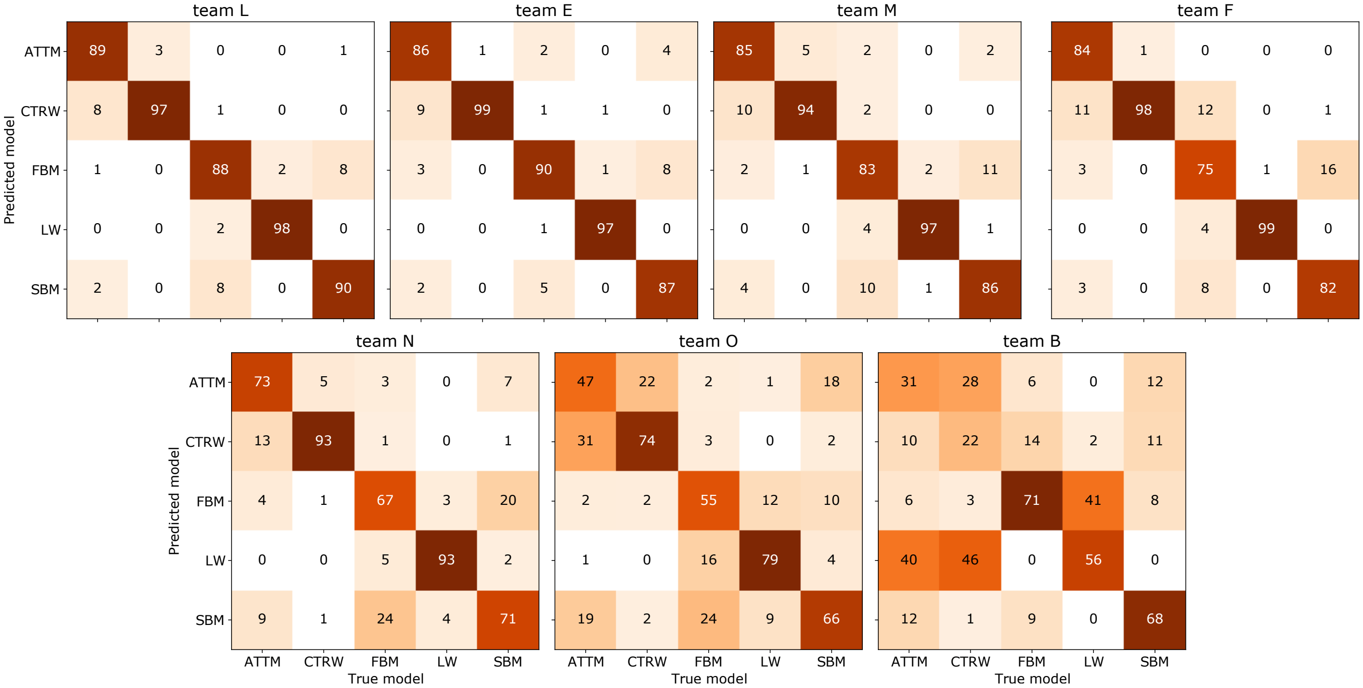

Supplementary Figure 13. **T2.3D methods' performance.** Confusion matrix of the ground truth model vs the predicted model for all the submitted methods for T2.3D. Teams are ordered according to to their ranking in the leaderboard. Numbers in matrix cells represent the number of correctly and incorrectly classified trajectories for each ground-truth model as percentages of the number of trajectories of the corresponding ground-truth model (column-based normalization). Thus, the percentages of correctly classified observations can be thought of as class-wise recalls.

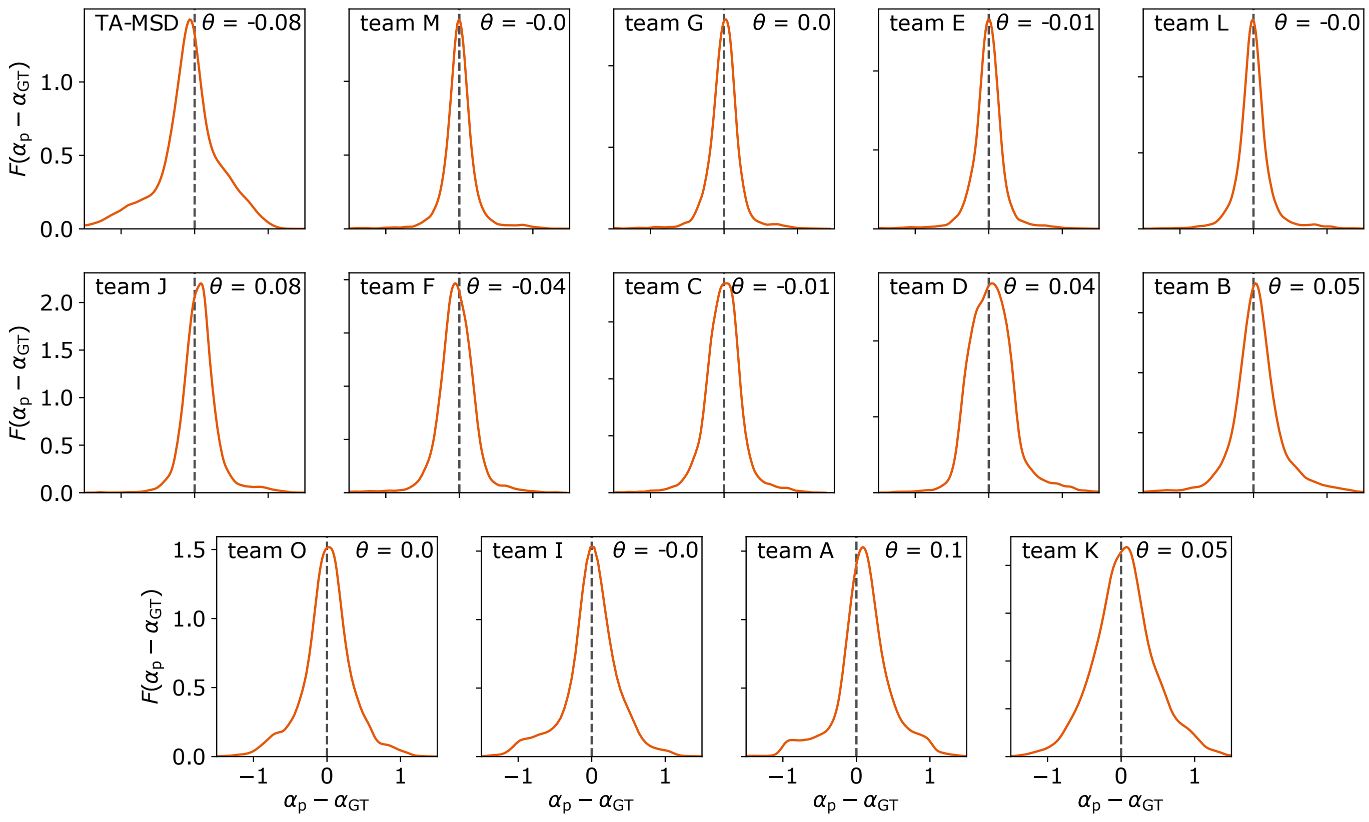

Supplementary Figure 14. **T1.1D prediction bias.** Empirical probability distributions of the difference between the predicted (α_p) and the ground-truth exponent (α_{GT}) for every method participating in T1.1D. The expectation value of the bias θ is reported in the plot. A dashed line representing the zero value is included as a guide-to-the-eye. Teams are ordered according to to their ranking in the leaderboard.

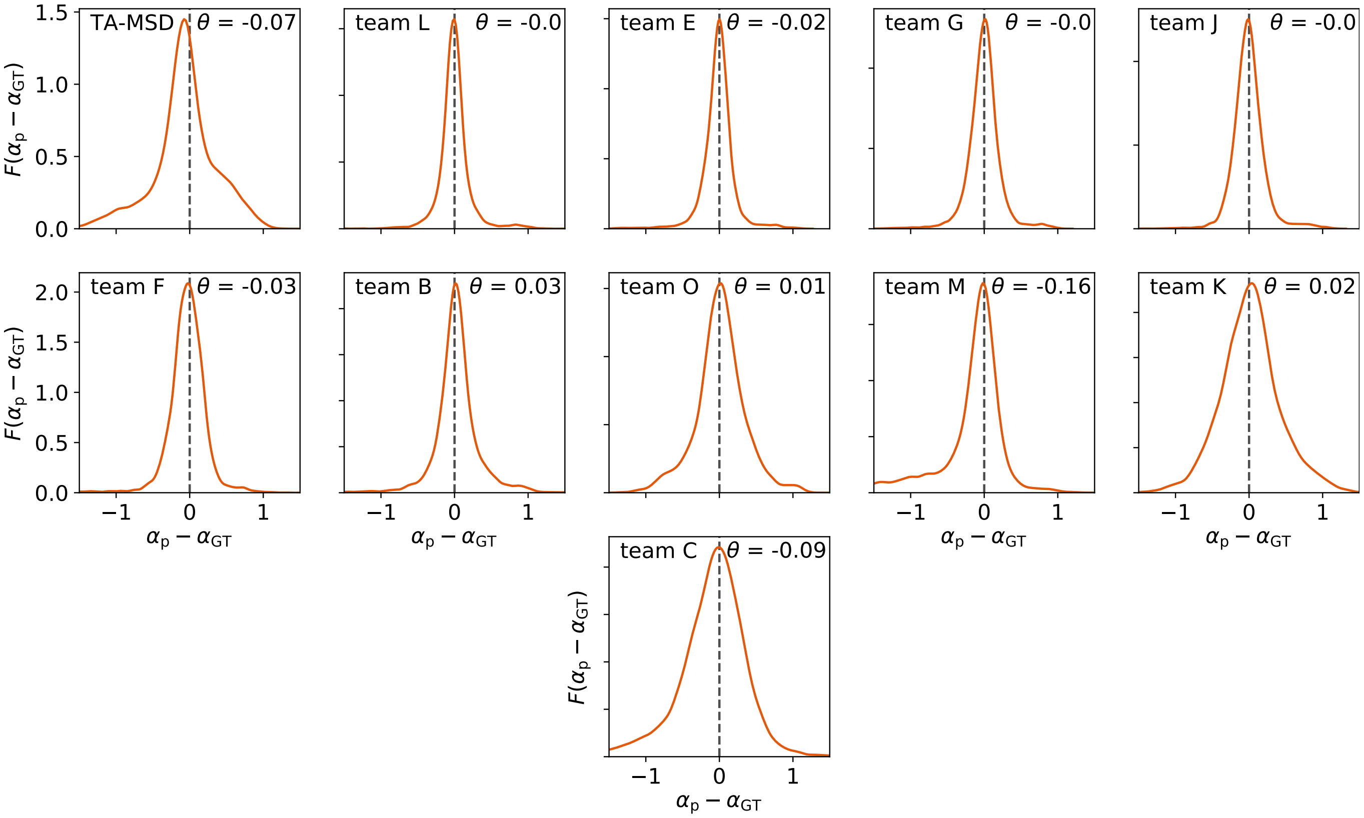

Supplementary Figure 15. **T1.2D prediction bias.** Empirical probability distributions of the difference between the predicted (α_p) and the ground-truth true exponent (α_{GT}) for every method participating in T1.2D. The expectation value of the bias θ is reported in the plot. A dashed line representing the zero value is included as a guide-to-the-eye. Teams are ordered according to to their ranking in the leaderboard.

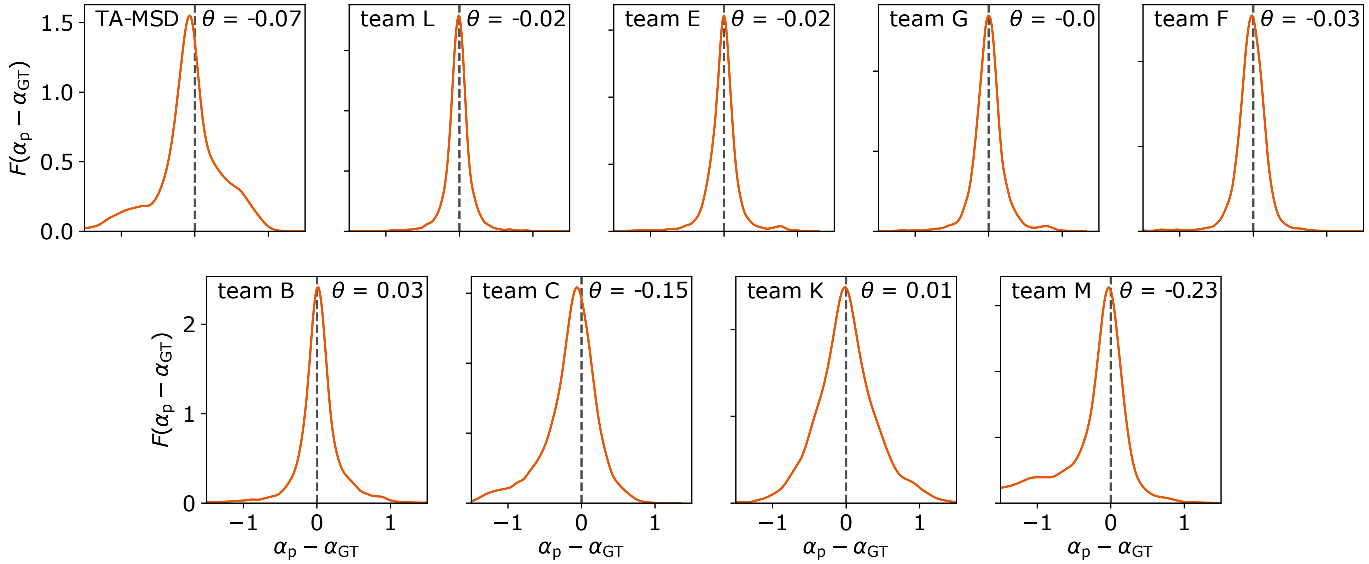

Supplementary Figure 16. **T1.3D prediction bias.** Empirical probability distributions of the difference between the predicted (α_p) and the ground-truth exponent (α_{GT}) for every method participating in T1.3D. The expectation value of the bias θ is reported in the plot. A dashed line representing the zero value is included as a guide-to-the-eye. Teams are ordered according to to their ranking in the leaderboard.

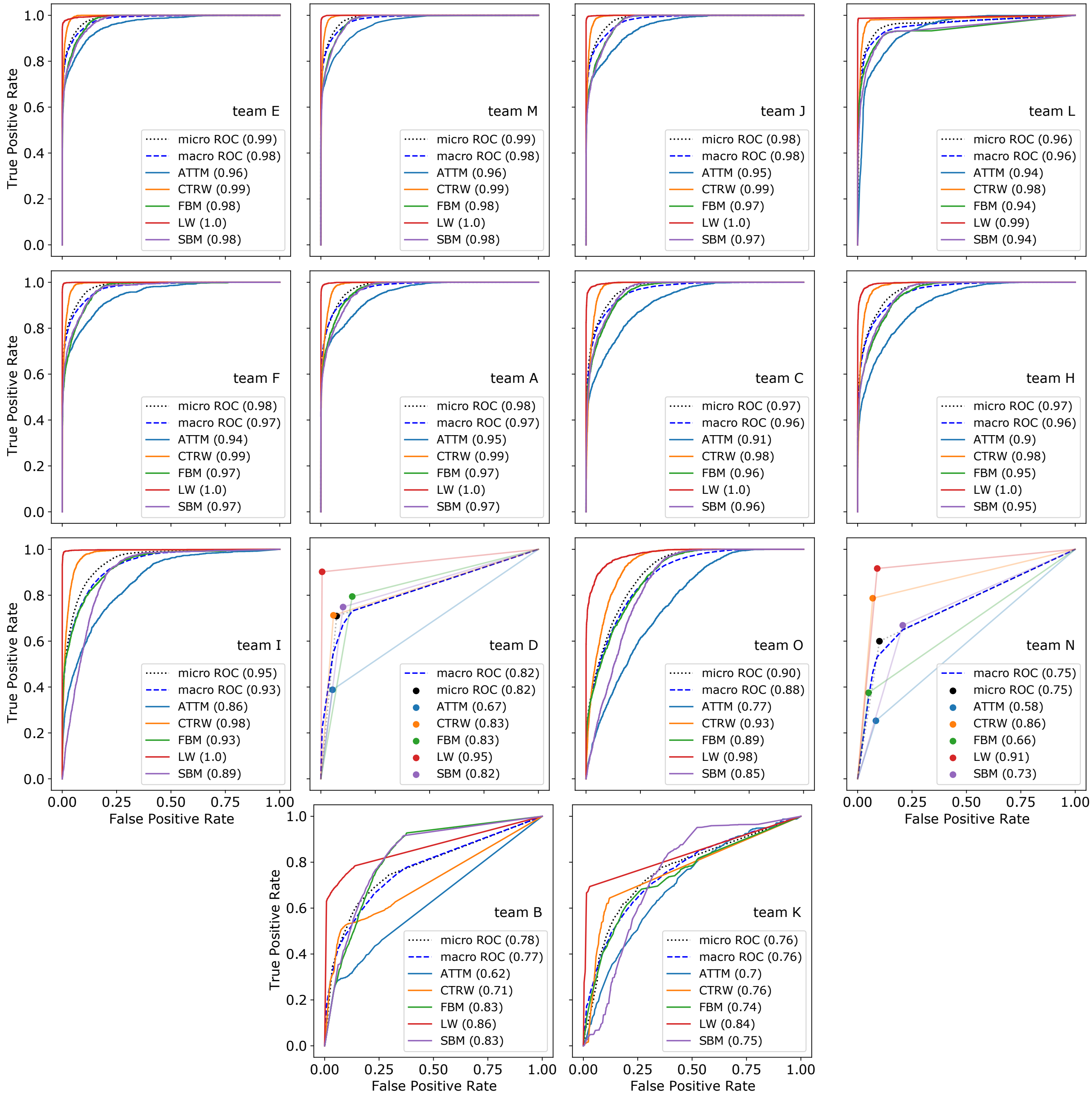

Supplementary Figure 17. **T2.1D ROC curves.** ROC curves obtained for each diffusion model, plus micro- and macro-average, for all the methods participating in T2.1D. AUC values are reported in the legend. Teams are ordered according to to their ranking in the leaderboard.

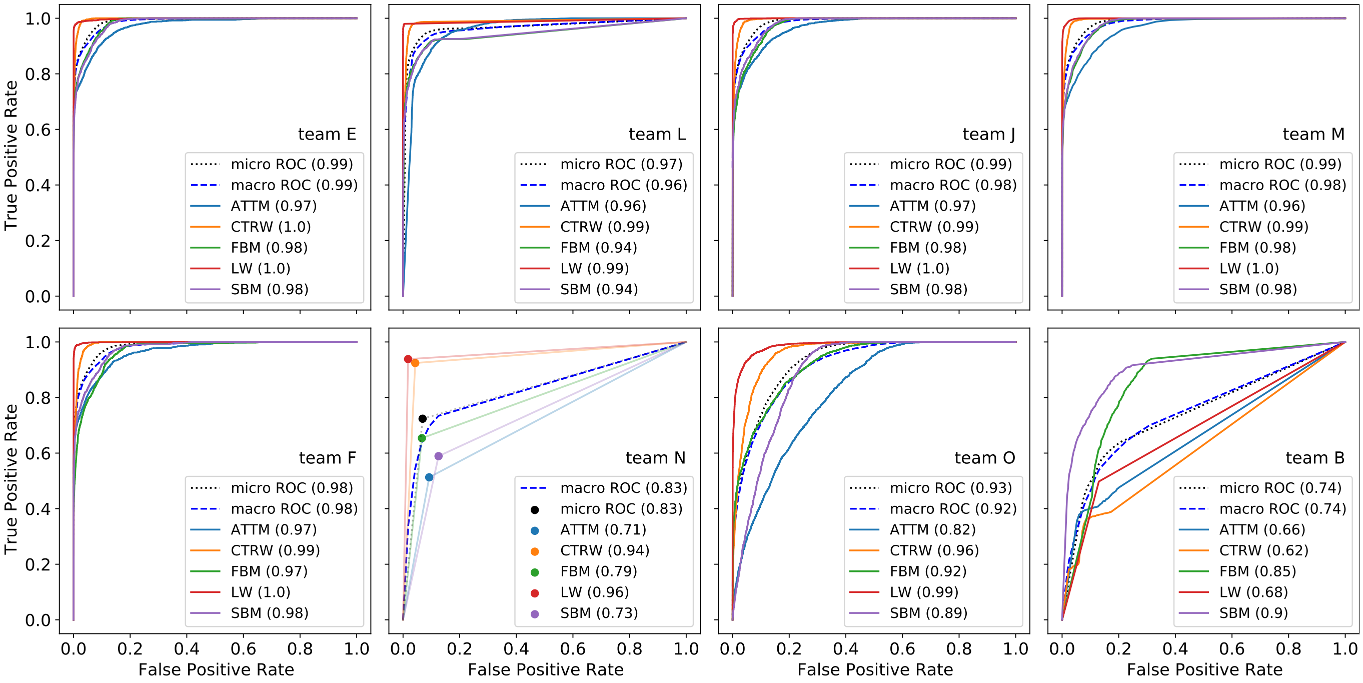

Supplementary Figure 18. **T2.2D ROC curves.** ROC curves obtained for each diffusion model, plus micro- and macro-average, for all the methods participating in T2.2D. AUC values are reported in the legend. Teams are ordered according to to their ranking in the leaderboard.

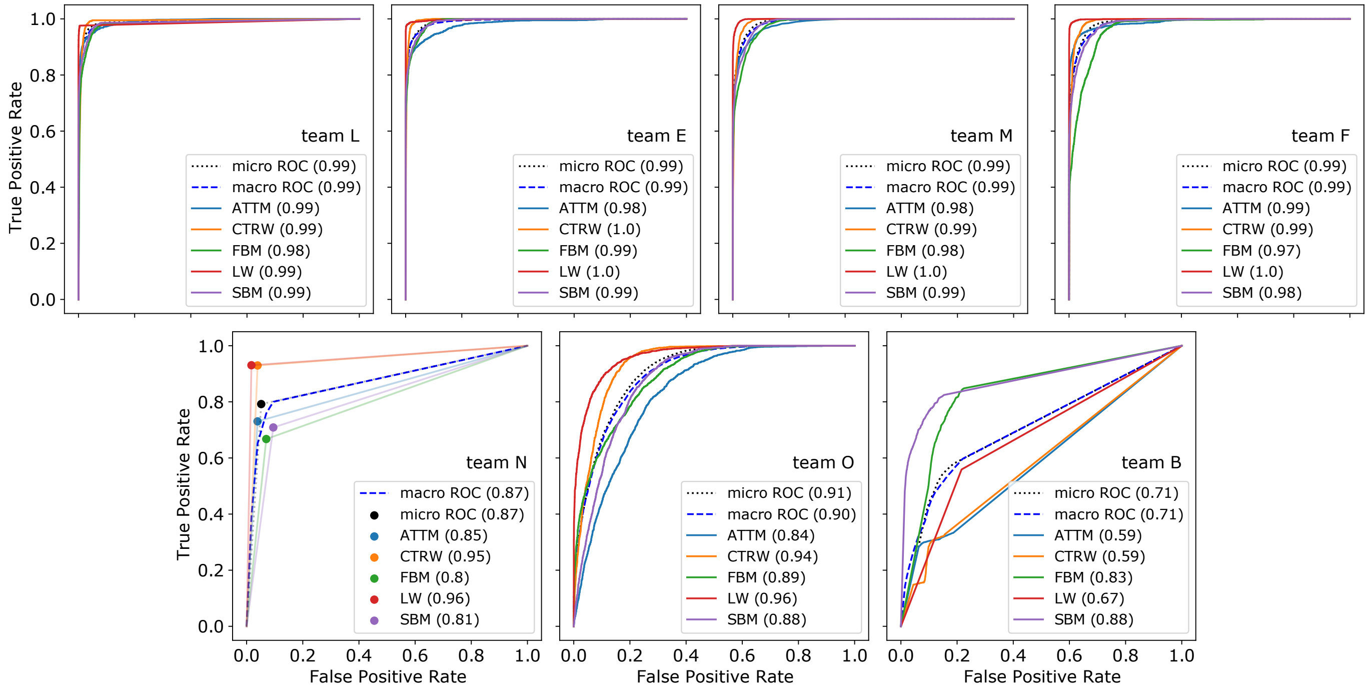

Supplementary Figure 19. **T2.3D ROC curves.** ROC curves obtained for each diffusion model, plus micro- and macro-average, for all the methods participating in T2.3D. AUC values are reported in the legend. Teams are ordered according to to their ranking in the leaderboard.

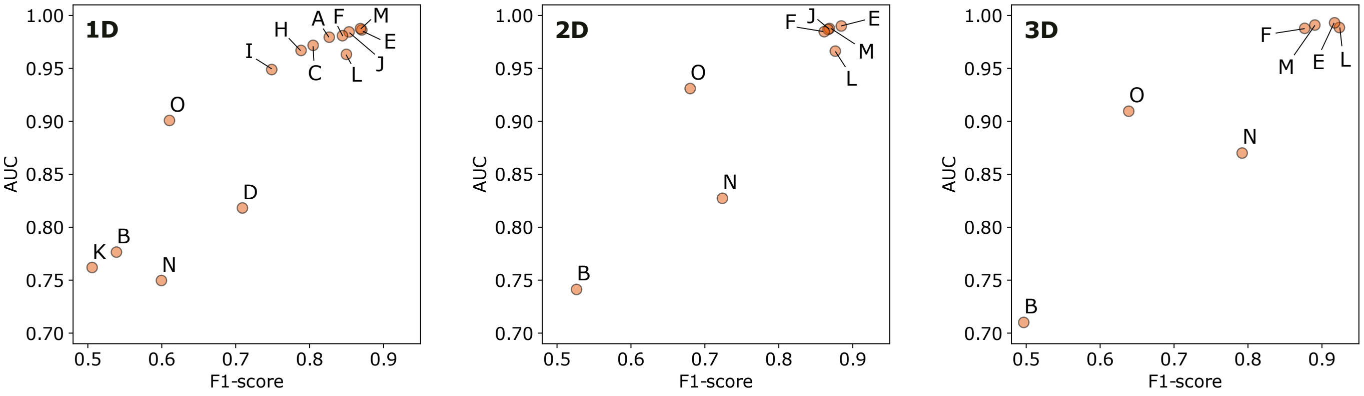

Supplementary Figure 20. **AUC vs F_1-score for T2.** Scatter plot of the micro-averaged AUC vs the F_1-score for all methods participating in T2.

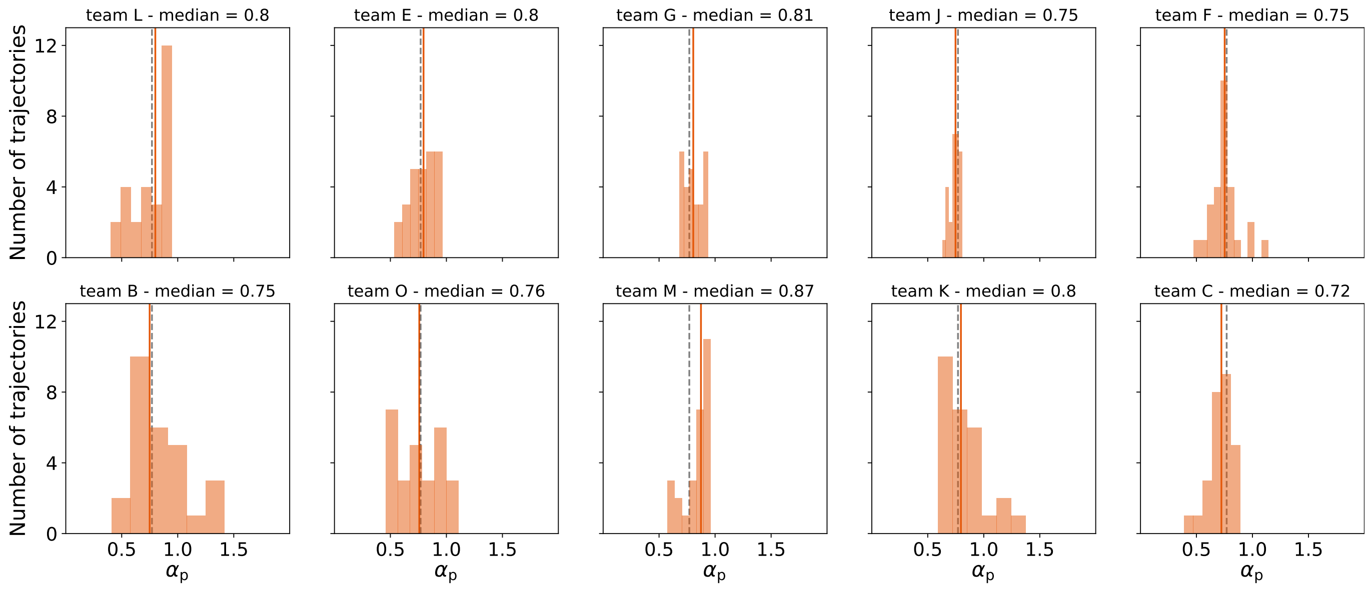

Supplementary Figure 21. **Prediction of anomalous diffusion exponent for experimental trajectories from Ref. [1].** Histogram of the anomalous diffusion exponent α_p predicted by all the methods participating in T1.2D. The continuous line represents the median value of α_p. The dashed line indicates the original estimation of α provided by Ref. [1].

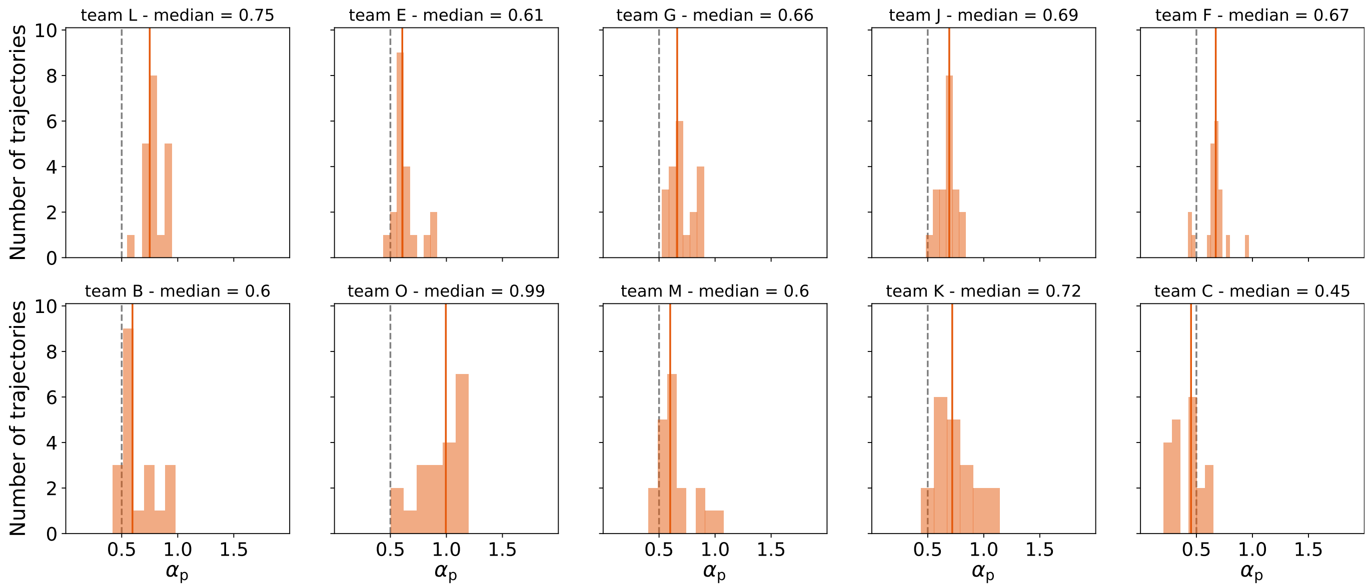

Supplementary Figure 22. **Prediction of anomalous diffusion exponent for experimental trajectories from Ref. [2, 3].** Histogram of the anomalous diffusion exponent α_p predicted by all the methods participating in T1.2D. The continuous line represents the median value of α_p. The dashed lines indicate the original estimation of α provided by Refs [2, 3].

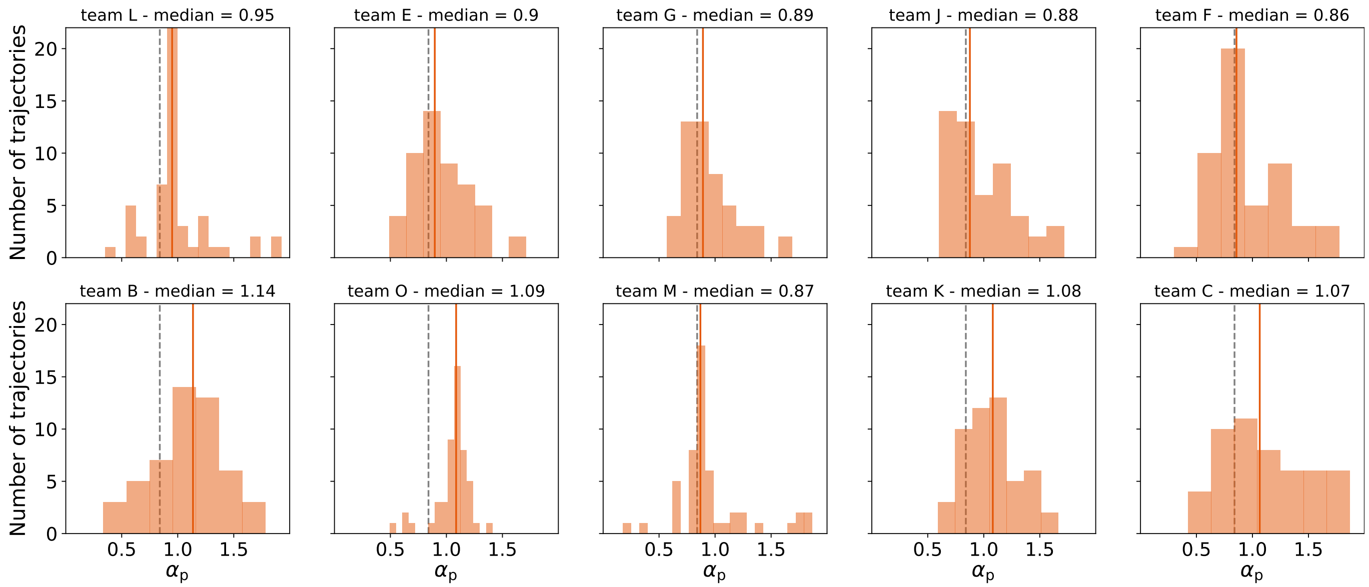

Supplementary Figure 23. **Prediction of anomalous diffusion exponent for experimental trajectories from Ref. [4].** Histogram of the anomalous diffusion exponent α_p predicted by all the methods participating in T1.2D. The continuous line represents the median value of α_p. The dashed line indicates the original estimation of α provided by Ref. [4].

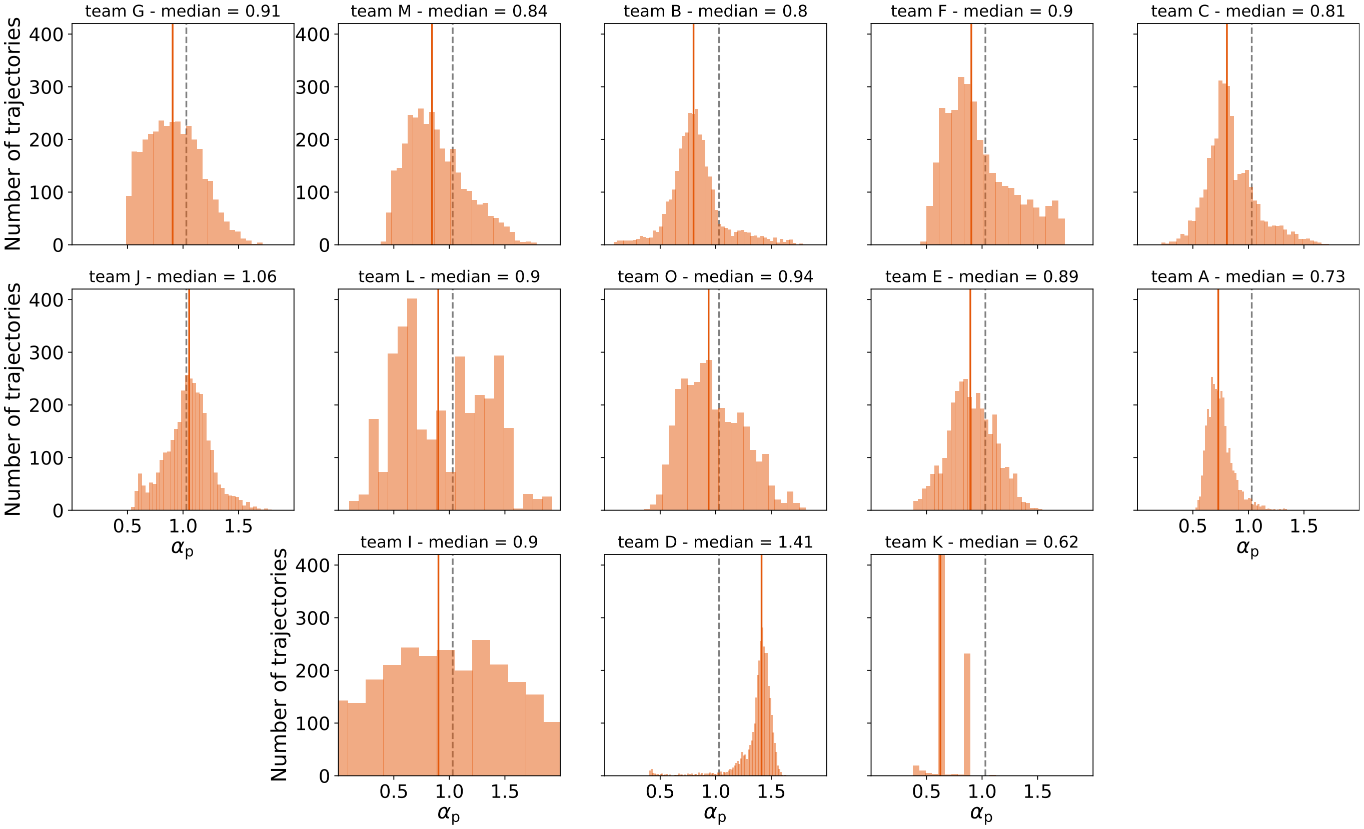

Supplementary Figure 24. **Prediction of anomalous diffusion exponent for experimental trajectories from Ref. [5].** Histogram of the anomalous diffusion exponent α_p predicted by all the methods participating in T1.1D. The continuous line represents the median value of α_p. The dashed line indicates the original estimation of α provided by Ref. [5].

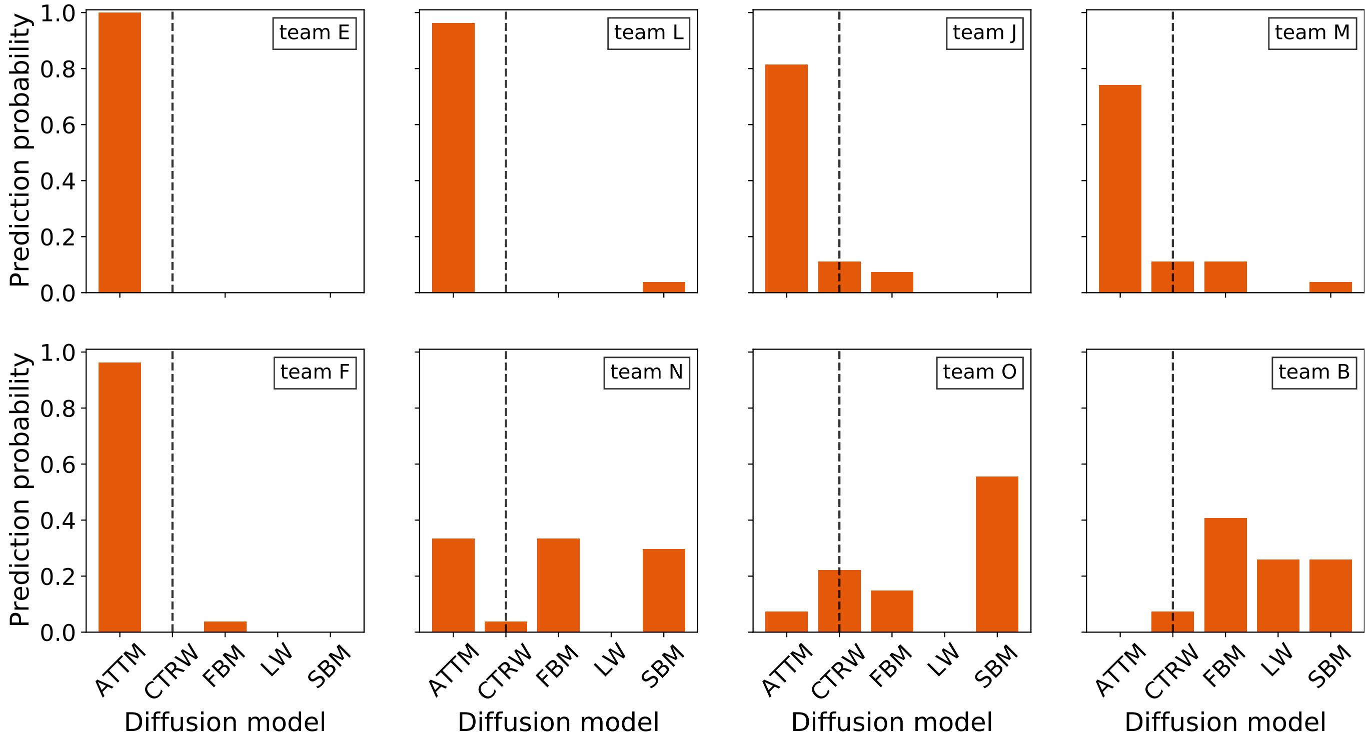

Supplementary Figure 25. **Prediction of diffusion model for experimental trajectories from Ref. [1].** Bar plot of the trajectory classification probability for the five anomalous diffusion model as predicted by all the methods participating in T2.2D. The dashed line indicates the original prediction of diffusion model provided by Ref. [1].

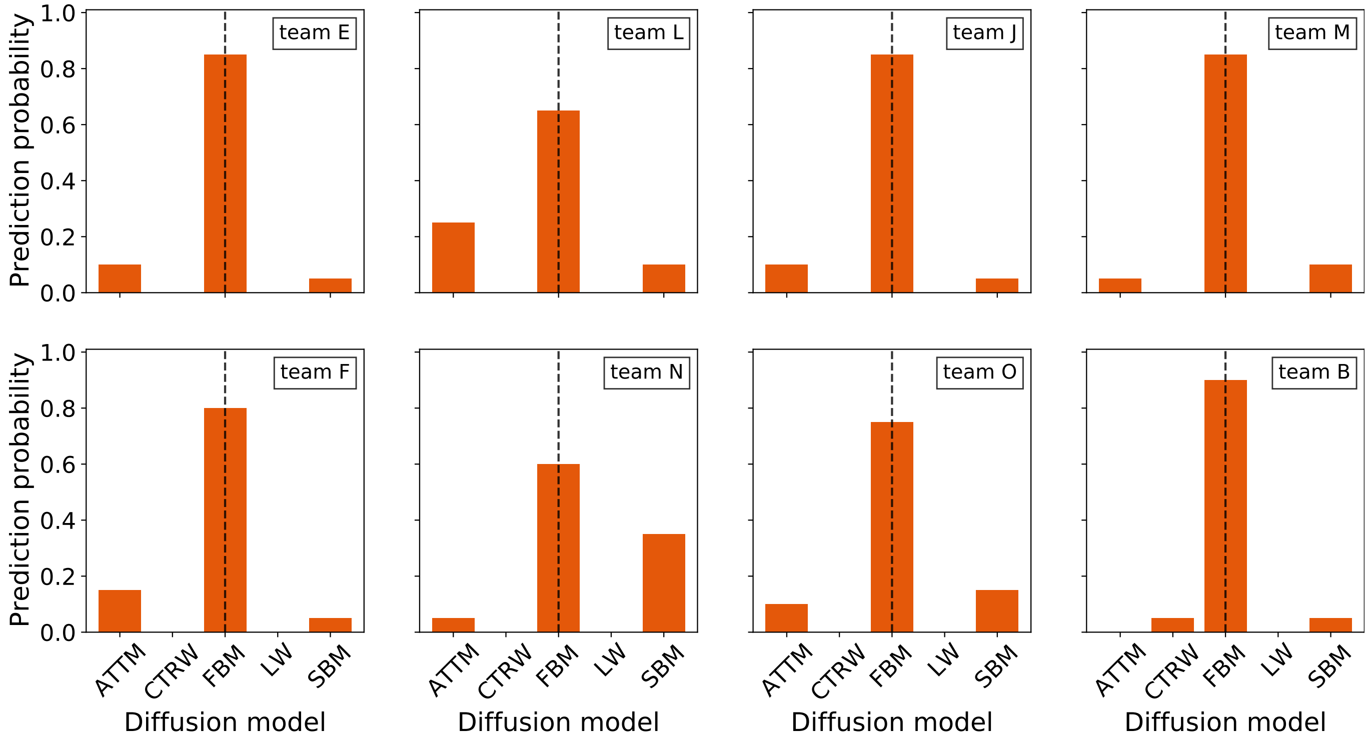

Supplementary Figure 26. **Prediction of diffusion model for experimental trajectories from Refs. [2, 3].** Bar plot of the trajectory classification probability for the five anomalous diffusion model as predicted by all the methods participating in T2.2D. The dashed line indicate the original prediction of diffusion model provided by Refs [2, 3].

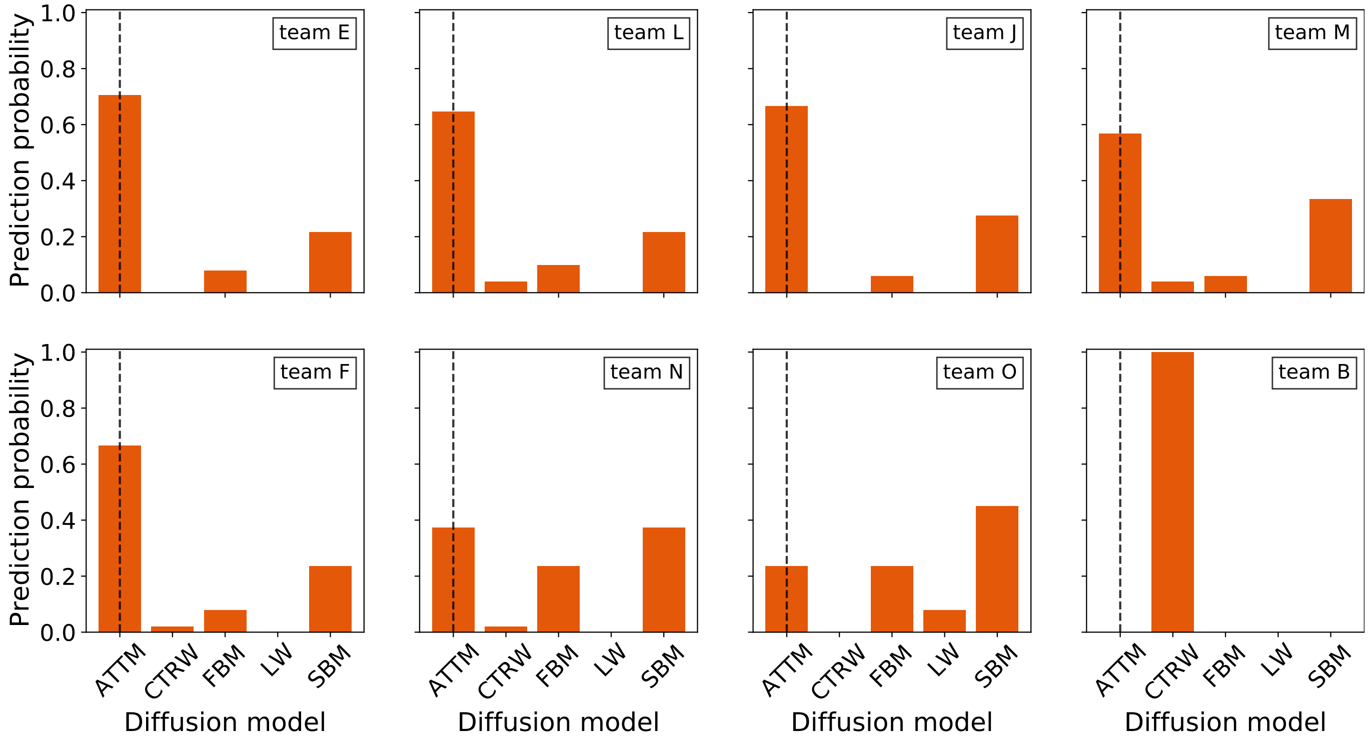

Supplementary Figure 27. **Prediction of diffusion model for experimental trajectories from Ref. [4].** Bar plot of the trajectory classification probability for the five anomalous diffusion model as predicted by all the methods participating in T2.2D. The dashed line indicates the original prediction of diffusion model provided by Ref. [4].

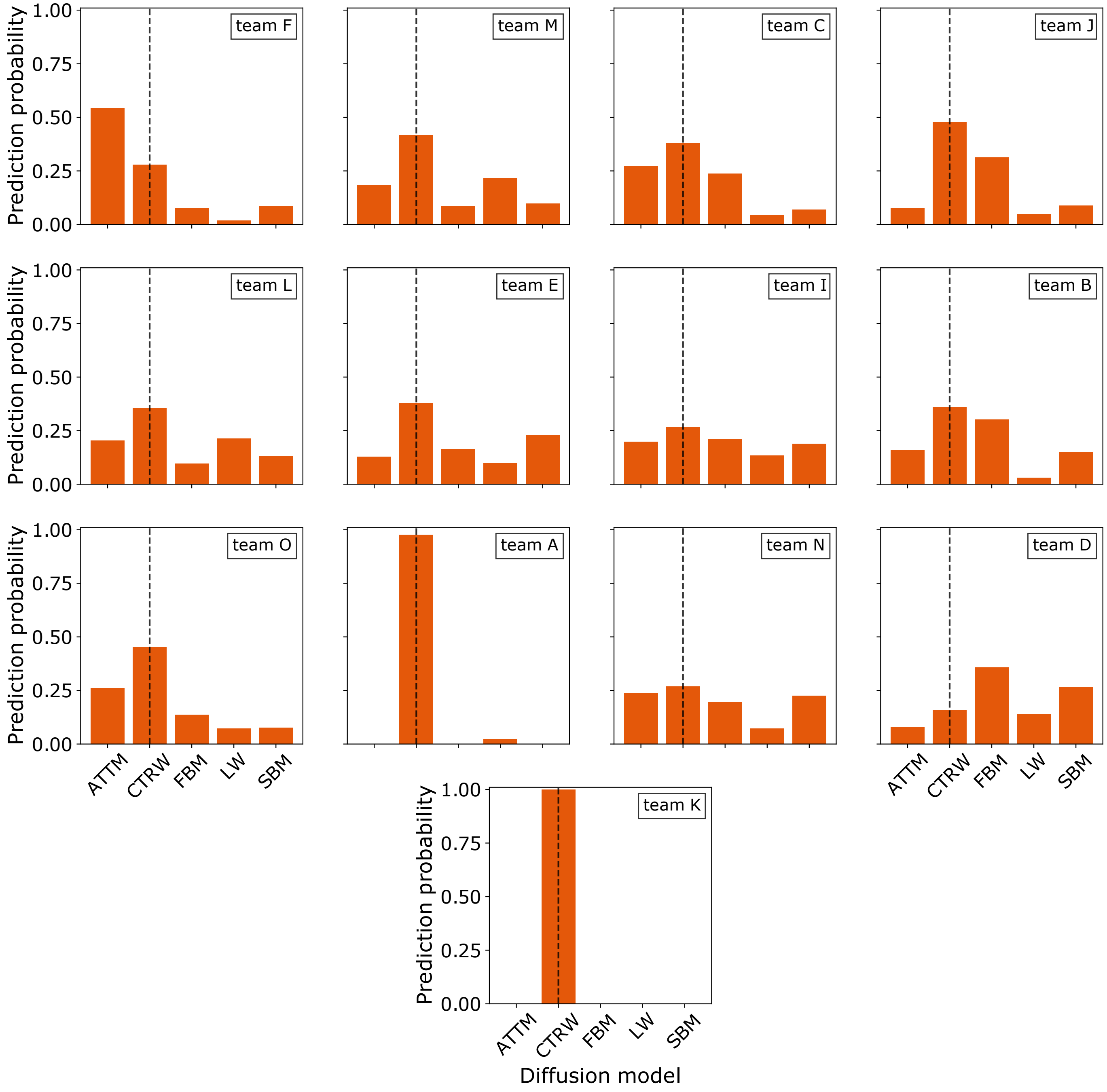

Supplementary Figure 28. **Prediction of diffusion model for experimental trajectories from Ref. [5].** Bar plot of the trajectory classification probability for the five anomalous diffusion model as predicted by all the methods participating in T2.1D. The dashed line indicates the original prediction of diffusion model provided by Ref. [5].

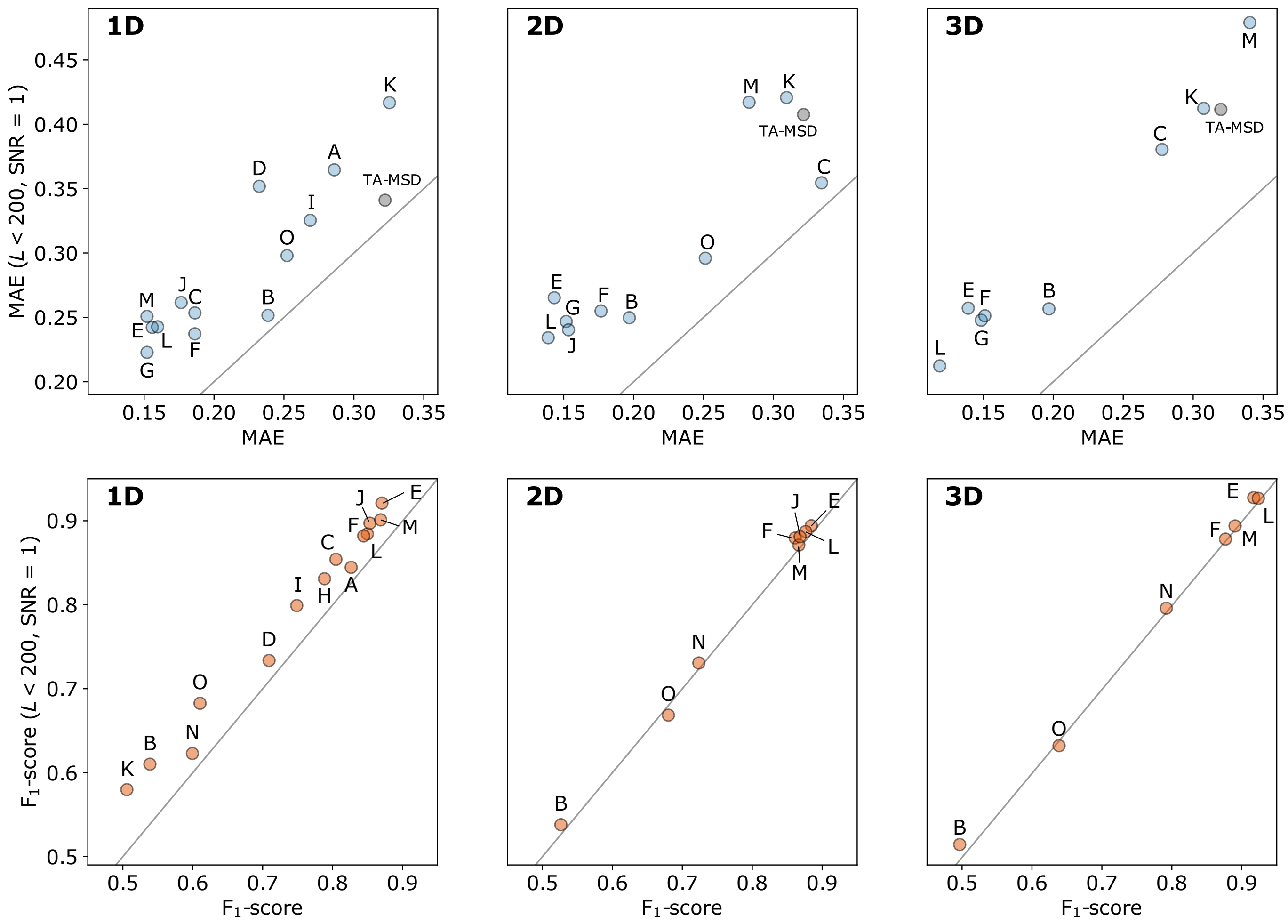

Supplementary Figure 29. **Metrics for short and noisy trajectories vs whole dataset.** Scatter plots of challenge metrics obtained over a subset of short and noisy trajectories ($L < 200$, SNR$= 1$) vs those obtained for the whole dataset for T1 (MAE, upper panels) and T2 (F_1-score, lower panels) in all the dimensions. Lines correspond to $y = x$, indicating equivalent performance on both datasets.

SUPPLEMENTARY NOTE 1: LIST OF TEAMS PARTICIPATING TO THE CHALLENGE

Team A: *Anomalous Unicorns*	
Contact:	Borja Requena<br>ICFO–The Institute of Photonic Sciences<br>Castelldefels (Barcelona), Spain
Reference:	Based on Refs. [6, 7]
Method:	HYDRAS (RNN + CNN)
Platform:	Python
Open-access:	https://github.com/BorjaRequena/AnDi-unicorns<br>https://github.com/AnDiChallenge/AnDi2020_TeamA_AnomalousUnicorns
Description:	Hydras are architectures that have a set of independent feature extractors (heads) that process the input trajectories. These all converge into a final set of fully connected layers (body) that process the output of the heads to perform inference. The feature extractors can be anything capable of processing trajectories of arbitrary lengths, such as recurrent neural networks (RNNs), convolutional neural networks (CNNs) or, even, other hydras. For T2, we have taken an ensemble of ten bi-headed hydras built with an RNN and a CNN as feature extractors. For T1, the resulting model is another ensemble of hydras that builds upon the result from T2. The resulting hydras have six heads: a hydra from T1 and five expert bi-headed hydras (RNN+CNN) that are trained to predict the anomalous exponent of a single diffusion model exclusively. This way, the body receives the output from all the model-specific feature extractors together with the opinion of the classifier. Each head is trained independently and then, in order to build the hydra, their weights are frozen while the body is trained. Finally, after a few epochs of body training, the head weights are unfrozen, and the entire hydra is trained with different learning rates: heads are trained with a much lower learning rate than the body. The entire source code can be found in the GitHub repository together with some examples.
Tasks:	T1.1D, T2.1D

Team B: *BIT*	
Contact:	Michael A. Lomholt<br>PhyLife, Department of Physics, Chemistry and Pharmacy, University of Southern Denmark<br>Odense M, Denmark
Reference:	[8, 9]
Method:	Bayesian inference
Platform:	Matlab
Open-access:	https://github.com/mlomholt/andi<br>https://github.com/AnDiChallenge/AnDi2020_TeamB_BIT
Description:	Bayesian inference using annealed importance sampling to sample from the posterior distribution. We attempted to use Bayes theorem to calculate the posterior probability distributions for the models and parameters. The likelihood functions, and to a large extent also the priors, could be derived from the descriptions and codes provided by the organizers. Effective Bayesian inference could be achieved for the SBM and FBM [8] models. However, the need to integrate out hidden waiting times impaired effective inference for ATTM, CTRW and LW. For ATTM and CTRW, we attempted to integrate out the waiting times together with the model parameters using Monte Carlo techniques. For LW, in 1D we used the forward algorithm on a hidden Markov model (but without including measurement noise) [9], while in 2D and 3D we used a goodness-of-fit test after inference with the other four models to exclude them, followed by a fit to the TA-MSD to obtain the anomalous diffusion exponent of the LW.
Tasks:	All

Team C: *DecBayComp*	
Contact:	Jean-Baptiste Masson<br>Institut Pasteur, Decision and Bayesian Computation lab<br>Paris, France
Reference:	[10]
Method:	Gratin: graphs on trajectories for inference
Platform:	Python
Open-access:	https://github.com/DecBayComp/gratin<br>https://github.com/AnDiChallenge/AnDi2020_TeamC_DecBayComp
Description:	First, each trajectory is turned into a graph, where nodes are the positions and edges connect positions following a pattern based on their time difference. Then, features computed from normalized positions are attached to nodes (e.g., cumulative distance covered since origin, distance to origin, maximal step size since origin). These graphs are then passed as input to a graph convolution module (graph neural network), which outputs, for each trajectory, a latent representation in a high-dimensional space. This fixed-size latent vector is then passed as input to task-specific modules, which can predict the anomalous exponent or the random walk type. Several output modules can be trained at the same time, using the same graph convolution module, by summing task-specific losses. The model can receive trajectories of any size as inputs. The high-dimensional latent representation of trajectories can be projected down to a 2D space for visualization and provides interesting insights regarding the information extracted by the model (see details in Ref. [10]).
Tasks:	T1.1D, T2.1D

Team D: *DeepSPT*	
Contact:	Taegeun Song<br>Center for AI and Natural Sciences, Korea Institute for Advanced Study<br>Seoul, Korea
Reference:	Based on Refs. [11, 12]
Method:	ResNet-MLP + XGBoost
Platform:	Python
Open-access:	https://github.com/TaegeunSONG/DeepSPT<br>https://github.com/AnDiChallenge/AnDi2020_TeamD_DeepSPT
Description:	We build our machine in the context of ensembles and hybrid structures. The applied preprocessing consists of three steps: 1) the noise is reduced by a 3-points moving average, 2) length of input trajectories are re-scaled to 100 points by a spline interpolation, and 3) the trajectories are normalized to the range $[0, 1]$. First, we prepare each normalized trajectory and extract user-defined features from the trajectory as an input for the ensemble modules. Then, we construct an ensemble of ten identical modules based on residual net (ResNet) [11] and multi-layer perceptron (MLP). The ResNet input is the normalized trajectory and the following MLP receives both an output of the ResNet and the prepared features. Finally, the ten outputs from the ResNet-MLP module are analyzed by a scalable tree boosting system (XGBoost) [12].
Tasks:	T1.1D, T2.1D

Team E: *eduN*	
Contact:	Stefano Bo<br>Max Planck Institute for the Physics of Complex Systems<br>Dresden, Germany
Reference:	[13]
Method:	RANDI (LSTM + dense NN)
Platform:	Python
Open-access:	https://github.com/booste/andi_for_organizers<br>https://github.com/AnDiChallenge/AnDi2020_TeamE_eduN
Description:	The method is based on recurrent neural networks (RNN). The RNN used in all tasks share the same basic architecture and differ only in the last layer or two. All the RNN have two long short-term memory (LSTM) layers (of dimension 250 and 50, respectively). For inference tasks (T1 and T3) the last output of the second LSTM layer is directly connected to the output layer. For classification tasks (T2 and T3), the last output of the second LSTM layer is followed by a dense layer including 20 nodes, which is then connected to the five dimensional output layer (representing each model with softmax activation).<br>We train multiple RNN that specialize in analyzing trajectories of a certain length. When presented with a trajectory of length l, we use the predictions of the two RNN trained on the nearest lengths (one on longer trajectories of length L_+ and one on shorter ones of length L_-) and weigh them according to their distance from l. For T1, we train 14 RNN for different lengths in 1D and 9 RNN for different lengths in 2D. For T2, we train 6 RNN for different lengths in 1D and 4 RNN for different lengths in 2D. In T3 all trajectories have the same length; we train 4 RNN: the first RNN to classify the model of the first segment, the second RNN to classify the model of the second segment, and two inference RNN; each inference RNN predicts the switching time, first exponent and the second exponent and their predictions are then averaged. We follow the same approach in 2D (but there we use a single RNN for the inference). We do not train RNN on 3D trajectories. For 3D data, we take projections on lower dimensions and use RNN trained on 2D and 1D data and average their outputs.<br>All RNN are trained using 3×10^6 trajectories that are generated using `andi-datasets` package [14]. To avoid overtraining, we split these trajectories in 30 datasets (each containing 10^5 trajectories) which are successively presented to the RNN. We use the first dataset to train for 5 epochs splitting it in batches of size 32. We then switch to another dataset, split it in batches of size 128 and train for 4 epochs. We repeat this procedure for 3 other datasets. We iterate the procedure using 5 datasets split into batches of size 512 each considered for 3 epochs and finally use 20 datasets split into batches of size 2048 for 2 epochs each. For memory reasons, we did not use the batches of size 2048 for trajectories containing large amounts of measurement, such as long or high-dimensional trajectories. We use recurrent dropout (20%) in both LSTM layers.<br>We preprocess the input data as follows: 1) We take the increment values of the trajectory. 2) We normalize the increments in a way that they have zero mean and unitary standard deviation for each trajectory. 3) To optimize the training, we re-shape the input trajectories into shorter trajectories of higher dimensions. For example, for the inference of 1D trajectories of length 225, the 224 increments are split into 56 blocks of dimension 4, $b_j = [\Delta x_{4j}, \Delta x_{4j+1}, \Delta x_{4j+2}, \Delta x_{4j+3}]$ with $j = 0, \ldots 55$. The chosen block size varies according to the trajectory length and dimension.
Tasks:	All

Team F: *Erasmus MC*	
Contact:	Hélène Kabbech<br>Erasmus MC, Department of Cell Biology<br>Rotterdam, The Netherlands
Reference:	Based on Ref. [15]
Method:	FEST
Platform:	Python
Open-access:	https://github.com/hkabbech/FEST_AnDiChallenge<br>https://github.com/AnDiChallenge/AnDi2020_TeamF_ErasmusMC
Description:	The Feature Extraction Stack long short-term memory (FEST) method was used to solve T1 and T2 and was applied to one-, two- and three-dimensional trajectory data. This method is divided in two parts: i) measurement of features at each point along the trajectories, and ii) training of a neural network consisting of a stack of bidirectional long short-term memory (LSTM) and fully connected ("Dense") layers [16].<br>The following features were computed: the displacements $\Delta\mathbf{r}_n(t) = (\Delta\mathbf{x}_n(t), \Delta\mathbf{y}_n(t), \Delta\mathbf{z}_n(t))$ of a particle between time t and $t+n$ (which is the difference between two particle positions $\mathbf{r}_t$ and $\mathbf{r}_{t+n}$, where $\mathbf{r}_t = (x_t, y_t, z_t)$ and $n \geq 1$) and the distances $d_n(t) = \sqrt{\Delta\mathbf{x}_n(t)^2 + \Delta\mathbf{y}_n(t)^2 + \Delta\mathbf{z}_n(t)^2}$. The features for 1D and 2D cases were similarly defined. Subsequently, a mean of distances between time $t-p$ and $t+p$, $\overline{d_{n,p}}(t)$, was calculated as $\overline{d_{n,p}}(t) = \frac{1}{2p+1}\sum_{k=t-p}^{t+p} d_n(k)$, where $p \geq 1$. All the mentioned features characterize how fast particles move. To gain information on the direction of motion, for 2D and 3D cases, the angles $\theta_n(t)$ between two displacement vectors $\Delta\mathbf{r}_n(t)$ and $\Delta\mathbf{r}_n(t-n)$ were computed.<br>The number of features that were used as input to the neural network depended greatly on the number of dimensions. For 1D case, only displacements could be computed, therefor we used $\Delta\mathbf{x}_n$, $n = \{1, 2\}$. Larger values of n led to smaller sizes of feature vectors. For 2D case, we computed six features: $\Delta\mathbf{x}_1$, $\Delta\mathbf{y}_1$, d_1, $\overline{d_{1,1}}$, $\overline{d_{2,1}}$ and θ_1. For 3D case, 6 other features were used: $\Delta\mathbf{x}_1$, $\Delta\mathbf{y}_1$, $\Delta\mathbf{z}_1$, d_1, $\overline{d_{1,1}}$, $\overline{d_{2,1}}$.<br>We built two similar neural network architectures for T1 and T2. Using the above-mentioned features, the output for T1 was a predicted value of α, and the outputs for T2 were probabilities of input track belonging to one of 5 diffusive models. The architectures of both neural network were built using functions from the Keras library [17]. In both cases, we used 3 bidirectional LSTM layers (with 2^6, 2^5 and 2^4 hidden nodes, respectively), followed by 4 Dense layers (with 2^5, 2^4, 2^3 and 1 (or 5) hidden nodes) with Dropout layers in between (with a dropout rate of 0.2 or 0.1). For T1, `ReLu` activation function was applied on each Dense layer, while for T2 `tanh` was applied with a `softmax` at the output layer. During the training, the models were optimized using the Adam optimizer and, as loss functions, we used the mean squared error (MSE) for T1 and categorical cross-entropy for T2.<br>The described networks had to be trained using trajectories with a fixed number of time points. For that, new datasets were created with the tool provided by the organizers (https://github.com/AnDiChallenge/ANDI_datasets [14]). To cover the variety of lengths that can be encountered in the challenge data, 4 different datasets were generated for each task, each consisting of different trajectory lengths: 50, 200, 400 or 600 time points. Thereby, each network was trained 4 times in order to create 4 distinct models. For each case (1D, 2D and 3D), we created 30000 tracks of length 50 for training and 6000 for validation (denoted 30000/6000) to keep a ratio 8:2, 7500/1500 trajectories of length 200, 3750/750 of length 400 and 2500/500 of length 600. Training and validation datasets were generated separately to ensure that all combined cases of α and diffusive models were present in both dataset.<br>The training have been carried out on a Linux system with a GPU GeForce GTX 1650 and a processor 2.60 GHz Intel 12 cores i7. An early stopping criterion was used to monitor the validation loss and prevent over-fitting. Finally, during the prediction phase and depending on the trajectory length, a combination of the different models was used to predict the outcome. Any track with a length below 100 was predicted with the model trained with 50 time points (denoted model50), any length falling between 100 and 300 with model200, between 300 and 500 with model400 and above 500 with model600. This approach would increase the accuracy of the prediction when the variety of trajectory length would be very diverse in a dataset.
Tasks:	T1, T2

Team G: *HNU*	
Contact:	Zihan Huang<br>School of Physics and Electronics, Hunan University<br>Changsha 410082, China
Reference:	[18]
Method:	Just LSTM it
Platform:	Python
Open-access:	https://github.com/huangzih/AnDi-Challenge<br>https://github.com/AnDiChallenge/AnDi2020_TeamG_HNU
Description:	The training dataset consisting of 1D trajectories is generated at 43 specific lengths (see the open-access link for details). The total size of training dataset is about 330 GB. Each trajectory is normalized before training so that its position's average and standard deviation are 0 and 1 respectively.<br>A long short-term memory (LSTM)-based recursive neural network (RNN) model is used to accomplish this competition task, where the dimension of the hidden layer is 64 and the number of stacked LSTM is 3. Models for each specific length are trained separately. 80% of training data is used for training, while the rest is used for validation. We implement the LSTM-based model by PyTorch 1.6.0. The model is trained with a batch size 512, where the loss function is the mean squared error (MSE). The optimizer is Adam with a learning rate $l = 0.001$. The learning rate is changed as $l \leftarrow l/5$ if the validation loss does not decrease for 2 epochs. When the number of such changes exceeds 1, the training process is early stopped to save time and avoid overfitting. The best epoch for a specific length is determined by the lowest mean absolute error (MAE) of the validation set.<br>The inference of challenge data is guided by the following rule: 1) If the original length of trajectory belongs to one of the 43 specific lengths, this trajectory will be directly used for inference. 2) Otherwise, a new length of this trajectory will be set as the closest smaller specific length. For instance, the new length of a trajectory with an original length 49 should be 45. The trajectory data is subsequently transformed into 2 sequences. For clarity, we set the trajectory data as $[x_1, x_2, \cdots, x_T]$, where T is the original length. We denote T_n as the new length with $T_n < T$. The two sequences are $[x_1, x_2, \cdots, x_{T_n}]$ and $[x_{T-T_n+1}, x_{T-T_n+2}, \cdots, x_T]$ respectively. Such two sequences are both used for inference, with model predictions α_1 and α_2. The predicted exponent α of the original trajectory is given by $\alpha = (\alpha_1 + \alpha_2)/2$.<br>To further improve the model performance, 5-Fold cross validation is utilized. However, due to the time limit of this competition, we only use a 3-fold average. On the other hand, by analyzing an external validation dataset containing 100000 1D trajectories, the predicted results for challenge data are multiplied by 1.011 and finally clipped to ensure reasonable predictions.<br>The methods for 2D and 3D tasks are both based on the solution for 1D trajectories. We separate the dimensions of the trajectories and treat the data of each dimension as 1D trajectories. Thus, we get predicted exponents α_x, α_y, and α_z for x, y, and z dimensions, respectively. The final results are $\alpha_{2D} = (\alpha_x + \alpha_y)/2$ for 2D trajectories, and $\alpha_{3D} = (\alpha_x + \alpha_y + \alpha_z)/3$ for 3D trajectories.
Tasks:	T1

Team H:*NOA*	
Contact:	Nicolás Firbas<br>Instituto Universitario de Matemática Pura y Aplicada, Universitat Politècnica de València<br>Valencia, Spain
Reference:	Based on Ref. [19]
Method:	Convolutional LSTM
Platform:	Python
Open-access:	https://github.com/NicoFirbas/ConvLSTM_AnDI<br>https://github.com/AnDiChallenge/AnDi2020_TeamH_NOA
Description:	The convolutional long short-term memory (convLSTM) approach combines convolutional neural networks (CNN) and long short-term memory networks (LSTM), similarly as described in Ref. [19]. An additional linear block placed after the LSTM uses the flattened LSTM output to predict the type of anomalous diffusion of the trajectory.<br>In more detail, it consists of a convolutional block (ConvBlock), a bidirectional LSTM, and a linear block (LinearOuts). The ConvBlock consists primarily of two one-dimensional convolutions with a filter size of two, each is followed by a ReLU. The first convolutional layer is more coarse and outputs 20 features, while the second layer takes the output of the first and outputs 64 features. At the end of the convolutional block, we have a dropout with dropout probability $p = 0.2$, to avoid overfitting, and a one-dimensional MaxPooling layer, which cuts the output size in half by selecting the larger of two adjacent entries. The bidirectional LSTM has three layers, each layer is followed by a dropout with probability of dropout $p = 0.2$. The final Block (LinearOuts) takes the flattened (2D tensor to 1D) output of the LSTM as its input and passes it to a fully connected linear layer, which has five output units that correspond to the five models used to produce the trajectories. The first two linear layers are followed by a ReLU activation and the final layer is not, as non-linearity is handled by an instance of nn.CrossEntropyLoss, during training, called the “criterion”.<br>Training of our method for the AnDi challenge was done using a hidden size of 32 and a learn rate of 0.001. However, later testing has shown that our model accuracy can be improved by increasing the hidden size to 128, while beyond that point we see a drop in accuracy. Training was performed by merging two data sets, which were generated with the `andi-datasets` package [14], the first of length 189810 and the second of length 150000. The resulting combined dataset was split into 75% training data and 25% test data. From the training data an additional 20% was reserved for validation data to be used by our early stopping algorithm. Our early stopping method saves the parameter state if there is an improvement in the mean validation loss, which is computed at the end of each epoch. We used 100 epochs and 10 patience for our early stopping.
Tasks:	T1.1D

Team I: *QuBI*	
Contact:	Carlo Manzo<br>Facultat de Ciències i Tecnologia, Universitat de Vic – Universitat Central de Catalunya (UVic-UCC)<br>Vic, Spain
Reference:	[20]
Method:	AnDi-ELM
Platform:	Matlab
Open-access:	https://github.com/qubilab/AnDi_ELM<br>https://github.com/AnDiChallenge/AnDi2020_TeamI_QuBI
Description:	Our model combines feature engineering and the use of an extreme learning machine (ELM). In brief, raw trajectories were first standardized to set their starting coordinates to zero and have a unitary standard deviation of displacements for $t_{\text{lag}} = 1$. For each $t_{\text{lag}} = 1, ..., 7$, two features were calculated, corresponding to $\frac{\log\langle\lvert x(t+t_{\text{lag}})-x(t)\rvert^k\rangle}{\log(t_{\text{lag}}+1)}$ for $k = 1, 2$. In addition, the correlation of absolute displacements obtained for $t_{\text{lag}} = 1$ was also included, for a total of 15 features per trajectory. Features were standardized using the z-score over the training dataset. The mean and standard deviation obtained for each feature of the training dataset was saved and later used to standardize the validation and test datasets. For a training dataset of n trajectories and f features with target values $\mathbf{T}$, the $n \times f$ feature matrix $\mathbf{X}$ is fed into a ELM composed by single hidden layer feedforward network (SLFN) with $m = 1000$ hidden nodes [21, 22]. A matrix of initial weights $\mathbf{W}$ of size $f \times m$ and a bias vector $\mathbf{b}$ of size $1 \times m$ are randomly initialized to connect observations to targets through:<br>$$f\left(\mathbf{XW} + \mathbf{ub}^{\mathbf{T}}\right)\mathbf{B} = \mathbf{HB} = \mathbf{T},$$<br>where $f(\cdot)$ represents the sigmoid activation function, $\mathbf{u}$ is a unitary vector of size $n \times 1$, and $\mathbf{B}$ is the matrix of output weight. The training of the SFLN is converted into solving an over-determined linear problem, whose least squares solution corresponds to the Moore-Penrose pseudoinverse of the hidden layer matrix $\mathbf{H}$ [21, 22]<br>$$\hat{\mathbf{B}} = \mathbf{H}^{\dagger}\mathbf{T}.$$<br>The SFLN was trained either as a regressor or as a classifier to provide predictions for T1 and T2 for 1D trajectories. Training was performed using only the dataset provided by the organizers (10000 trajectories per subtask) during the Development phase of the challenge. Training took typically 5 seconds on a MacBookPro with a 8-Core Intel Core i9 processor with 2.4GHz speed.
Tasks:	T1.1D, T2.1D

Team J: *FCI*	
Contact:	Tom Bland<br>The Francis Crick Institute<br>London, UK
Reference:	Based on Refs. [23, 24]
Method:	CNN
Platform:	Python
Open-access:	https://github.com/tsmbland/andi_challenge<br>https://github.com/AnDiChallenge/AnDi2020_TeamJ_FCI
Description:	We use a convolutional neural network structure adapted from the models used in Refs. [23, 24]. For T1 and T2, this consists of a series of convolutional blocks, followed by a global max-pooling layer over the temporal dimension, which feeds into a dense network. For T1, the model outputs a single number representing the predicted anomalous exponent. For T2, the model outputs 5 numbers, representing a probability (from 0-1) for each diffusion type. For T3, convolutional blocks are followed by a 1×1 convolutional network, which outputs an array of size $(1, n)$, where n is the number of steps in the trajectory, representing the probability of a switch at each position in the trajectory. The same network architectures can be used in 1D and higher dimensions, varying only the number of input features. Models were built using TensorFlow in Python, and code is available on Github.<br>Training data were generated using the `andi-datasets` package [14]. Trajectories were first preprocessed by taking the difference between successive positions, and normalized by dividing by the mean step size. For T1 and T2, a single model was simultaneously trained on trajectories of all lengths (ranging from 5-1000 steps). To permit mini-batch gradient descent with tracks of variable length, shorter tracks within each batch were padded with zeros to ensure a consistent input size (Note: padding is only necessary during training, and inference can be carried out with or without padding). For T3, training data consisted of trajectories 200-steps in length with a single changepoint, as per the challenge, but the method could be adapted to variable trajectory lengths and multiple changepoints.<br>For all models, training was carried out with a batch size of 32 and an Adam optimizer with a learning rate of 0.001, until a performance plateau was reached (up to a maximum of 1.28 million trajectories, with each trajectory seen by the networks only once).
Tasks:	T1.1D, T1.2D, T2.1D, T2.2D, T3.1D, T3.2D

Team K: *TSA*	
Contact:	Erez Aghion<br>Max Planck Institute for the Physics of Complex Systems<br>Dresden, Gemany
Reference:	[25]
Method:	Scaling analysis, and feature engineering (for T2)
Platform:	Python
Open-access:	https://github.com/ErezAgh/ANDI-challange-codes-<br>https://github.com/AnDiChallenge/AnDi2020_TeamK_TSA
Description:	This approach is based on theory, as opposed to pure data-driven methods. Anomalous diffusion can be described via more than just the Hurst exponent. The assumptions of the central limit theorem, which leads to standard diffusion, can be violated in three distinct ways: Increment correlations (like in FBM), fat-tailed increment distribution (like in CTRW), and nonstationarity of the increments' distribution, like in SBM. Each of these three paths can be characterized by its own scaling exponent, and can be measured directly in data, using methods of time-series analysis. The exponent J, describing the first violation, can be measured, e.g., using detrended fluctuations analysis. The exponent L, for the second violation, is measured from the temporal scaling of the time-average of the squared increments of the process. Finally, The exponent M is measured from the scaling of the time-average of the increments' absolute value. These exponents can be measured in any number of dimensions. Their sum leads to the Hurst exponent: $H = M + L + J - 1$ [25–27]. To estimate the Hurst exponent for T1, we evaluate J, L and M using methods which were specifically adapted for noise filtering. Importantly, this approach is not model-dependent, and our algorithm can be applied also to other types of data, not generated by one of the five models in the AnDi challenge.<br>For T2, we construct a small set of educated questions, targeted to characterize different properties of the paths in the data set, via precise analysis of the increments of the process. When comparing between various models outside of the AnDi challenge, here we would need to construct a new set of questions for the new models. Some of the questions are aimed for various general relations between the three exponents described above, others, to more specific properties of the individual types of paths involved in the challenge. The answers of each question can be “yes" (= 1) or “no" (= 0) (or “maybe" (= 2)). An example for a question about the exponents: Is $(J - 0.5) > (M - 0.5) + (L - 0.5)$? Namely, is the effect of autocorrelations on the Hurst exponent stronger than the combined effect of the increment distribution? This question separates between FBM and LW on the one side, and ATTM and SBM on the other. An example for a question beyond the exponents, is given by the comparison of the autocorrelations of the increments of the process, versus that of their absolute value. This question is highly selective for distinguishing Lévy walk from all the others. For each trajectory in the competition data set: we generate a set of answers using the same algorithm, and then generate an array of probabilities for this set to be either ATTM, CTRW, FBM, LW, or SBM. This is done by counting how many times a similar line of answers appeared in the training set for each type of process, divided by the total number of occurrences. The answer is, e.g.: $[0.125; 0.025; 0.85; 0.0; ...]$. The larger the training set, the more accurate is the evaluation of the probabilities. If a new set of answers is not found in the training file, a reduced number of selected questions are asked again, making the choice less selective. The selectivity of the questions, and the time-series analysis techniques used, also affect the quality of the final results. This method is similar in one and higher dimensions.
Tasks:	T1, T2.1D

Team L: *UCL*	
Contact:	Giorgio Volpe<br>Department of Chemistry, University College London<br>London, UK
Reference:	[28]
Method:	CONDOR
Platform:	Matlab
Open-access:	https://github.com/sam-labUCL/CONDOR<br>https://github.com/AnDiChallenge/AnDi2020_TeamL_UCL
Description:	Our method named Classifier Of aNomalous DiffusiOn tRajectories (CONDOR) relies on at first analyzing the trajectories to extract features (and their statistics) such as the trajectory length, velocity (with sign and absolute value, different sampling rates), rate of variation, Fourier Transform, Power Spectral Density, autocorrelation function, time-averaged MSD, and wavelet transform, among others. This analysis is performed on each dimension separately.<br>T2: These features are the inputs for a deep feed-forward neural network (5 categories, 2 hidden layers, 20 neurons per layer, trained with a 10^5 trajectory dataset) which classifies the model. The classification is then reprocessed in order by two similar neural networks (3 categories and 2 categories, instead of 5) that improve the precision on distinguishing among ATTM, FBM and SBM or between ATTM and CTRW, respectively. The combination of these three networks is our predictor for T2.<br>T1: To estimate α, we use the arithmetic average of the outputs of two different methods based on neural networks. Briefly, in a first method, the result of the classification (T2) is added as an input to the list of features above. These new features become the inputs for a 1×4 tree of networks (2 hidden layers, 20 neurons, trained with 3e5 trajectory datasets), where the parent network has 4 equally spaced α categories (in the range 0.05 to 2). Each of these categories is then branched into a different network with 5 equally spaced α categories in the corresponding α range. The (overestimated) predicted value of α is the average value in that category. In a second method, the result of the classification is not used as an input but is used to split the data into 5 categories each one analyzed by a different network (architecture and training as above). In particular, the networks for ATTM and CTRW have 5 α categories in the range 0.05 to 1. The network for LW has 5 α categories in the range 1 to 2. Finally, the prediction for FBM and SBM is based on a 1×2 tree of networks with the parent network having 2 equally spaced categories in the range 0.05 to 2, each then refined by a 5-category network in the corresponding range. The (underestimated) predicted value of α is the average value of the corresponding α range.
Tasks:	T1, T2

Team M: *UPV-MAT*	
Contact:	Òscar Garibo i Orts<br>Instituto Universitario de Matemática Pura y Aplicada, Universitat Politècnica de València<br>Valencia, Spain
Reference:	[29]
Method:	Recurrent neural networks for trajectory profiling
Platform:	Python
Open-access:	https://github.com/OscarGariboiOrts/ANDI_Challenge<br>https://github.com/AnDiChallenge/AnDi2020_TeamM_UPV-MAT
Description:	We have defined a recurrent neural network (RNN) architecture based on convolutional layer to feature extraction, bidirectional long short-term memory (LSTM) to learn the characteristics of the trajectory and Dense layers to smooth the signal to the final result. For T1, we have followed the same approximation, but building up to 12 different models for trajectories of different length. We have built models for trajectories in the length intervals: $[10, 20]$, $(20, 30]$, $(30, 40]$, $(40, 50]$, $(50, 100]$, $(100, 200]$, $(200, 300]$, $(300, 400]$, $(400, 500]$, $(500, 600]$, $(600, 800]$, and $(800, 1000]$, thus checking each trajectory length and applying the proper model.
Tasks:	T1, T2

Team N: *Wust ML A*	
Contact:	Janusz Szwabiński<br>Faculty of Pure and Applied Mathematics, Hugo Steinhaus Center, Wrocław University of Science and Technology,<br>Wrocław, Poland
Reference:	Based on Refs. [30, 31]
Method:	RISE for 1D - MrSEQL for 2D and 3D
Platform:	Python
Open-access:	https://github.com/szwabin/ANDI-challenge/<br>https://github.com/AnDiChallenge/AnDi2020_TeamN_WustMLA
Description:	RISE makes use of several series-to-series feature extraction transformers (fitted auto-regressive coefficients, estimated autocorrelation coefficients, power spectrum coefficients), which provide data to build a time series forest classifier. MrSEQL converts the numeric time series vector into strings to create multiple symbolic representations of the time series. The symbolic representations are then used as input for a sequence learning algorithm, to select the most discriminative subsequence features for training a classifier using logistic regression.
Tasks:	T2

Team O: *Wust ML B*	
Contact:	Hanna Loch-Olszewska & Patrycja Kowalek<br>Faculty of Pure and Applied Mathematics, Hugo Steinhaus Center, Wrocław University of Science and Technology,<br>Wrocław, Poland
Reference:	Based on Refs. [32–34]
Method:	Gradient boosting regression and classification
Platform:	Python
Open-access:	https://github.com/HannaLochOlszewska/ANDI_challenge<br>https://github.com/pkowalek/ANDI-challenge<br>https://github.com/AnDiChallenge/AnDi2020_TeamO_WustMLB1<br>https://github.com/AnDiChallenge/AnDi2020_TeamO_WustMLB2
Description:	Our approach is related to the feature-based methods described in Refs. [32–34], with an extended list of features used for extraction of the trajectories' characteristics. We used the gradient boosting algorithm in XGBoost (T1) and Gradient Boosting (T2) architectures. Such procedures allow us to examine trajectories with different lengths by extracting characteristics such as diffusion coefficient, anomalous diffusion exponent, fractal dimension, or gaussianity. The full set of features is listed in the Github repository. Each task and dimension gets a different set of features, depending on the problem behind the task. Both algorithms (Gradient Boosting, XGBoost) belong to the class of ensemble learning, i.e., methods that generate many base classifiers/regressors (decision trees in this case) and aggregate their results. We decided to use these classifiers as the idea behind the classifiers is easy to understand and interpret. The training was performed on 70000 trajectories generated using `andi-datasets` package [14] (for each task and subtask). Each set was balanced with respect to the anomalous exponent value (T1) or the model (T2).
Tasks:	T1.1D, T1.2D, T2

SUPPLEMENTARY NOTE 2: DETAILS OF EXPERIMENTS

Label : *GC*	
Reference:	[1]
Tracer:	mRNA molecules
Environment:	Cytosol of *E. Coli*
Dimension:	2D projection of a 3D movement
Experimental details:	The mRNA detection system consists of the bacteriophage MS2 coat protein fused to green fluorescent protein (GFP), and a reporter RNA containing 96 tandemly repeated MS2- binding sites.
Number of trajectories:	54
Trajectory length:	140 to 1628 frames
Frame rate:	1 frame/s
Localization precision:	NA

Label : W_{A}	
Reference:	[2, 3]
Tracer:	Telomeres
Environment:	Nucleus of bone osteosarcoma cells (U2OS, DSMZ-No.ACC785)
Dimension:	2D projection of a 3D movement
Experimental details:	GFP-tagged TRF-2 construct that recognizes the human telomeric sequences TTAGGG.
Number of trajectories:	200
Trajectory length:	500 frames
Frame rate:	5 frame/s
Localization precision:	18 nm

Label : *M*	
Reference:	[4]
Tracer:	DC-SIGN receptor
Environment:	Plasma membrane of Chinese hamster ovary cells
Dimension:	2D
Experimental details:	DC-SIGN receptors were labeled through half-antibody fragments conjugated to quantum dots.
Number of trajectories:	109
Trajectory length:	182 to 2000 frames
Frame rate:	60 frame/s
Localization precision:	$\approx$ 20 nm

Label : *Wi*	
Reference:	[5]
Tracer:	Caesium atoms
Environment:	Optical lattice
Dimension:	1D
Experimental details:	The atoms are radially confined by a running wave optical trap. Axially the atoms are trapped within the sites of the lattice formed by two counter-propagating laser beams. During the experimental sequence, only the lattice potential is lowered, while the radial confinement is held constant at all times. This allows one to limit the diffusion of the atoms along the lattice axis, justifying an effective one-dimensional description.
Number of trajectories:	3331
Trajectory length:	$\approx$ 10 frames
Frame rate:	2 frame/s
Localization precision:	2 μm

SUPPLEMENTARY REFERENCES

[1] I. Golding and E. C. Cox, Physical nature of bacterial cytoplasm, Physical review letters **96**, 098102 (2006).
[2] L. Stadler and M. Weiss, Non-equilibrium forces drive the anomalous diffusion of telomeres in the nucleus of mammalian cells, New Journal of Physics **19**, 113048 (2017).
[3] D. Krapf, N. Lukat, E. Marinari, R. Metzler, G. Oshanin, C. Selhuber-Unkel, A. Squarcini, L. Stadler, M. Weiss, and X. Xu, Spectral content of a single non-Brownian trajectory, Physical Review X **9**, 011019 (2019).
[4] C. Manzo, J. A. Torreno-Pina, P. Massignan, G. J. Lapeyre Jr, M. Lewenstein, and M. F. G. Parajo, Weak ergodicity breaking of receptor motion in living cells stemming from random diffusivity, Physical Review X **5**, 011021 (2015).
[5] F. Kindermann, A. Dechant, M. Hohmann, T. Lausch, D. Mayer, F. Schmidt, E. Lutz, and A. Widera, Nonergodic diffusion of single atoms in a periodic potential, Nature Physics **13**, 137 (2017).
[6] G. Muñoz-Gil, C. Romero, N. Mateos, L. I. de Llobet Cucalon, M. Beato, M. Lewenstein, M. F. Garcia-Parajo, and J. A. Torreno-Pina, Phase separation of tunable biomolecular condensates predicted by an interacting particle model, bioRxiv (2020).
[7] D. H. Wolpert, Stacked generalization, Neural networks **5**, 241 (1992).
[8] J. Krog, L. H. Jacobsen, F. W. Lund, D. Wüstner, and M. A. Lomholt, Bayesian model selection with fractional Brownian motion, Journal of Statistical Mechanics: Theory and Experiment **2018**, 093501 (2018).
[9] S. Park, S. Thapa, Y. Kim, M. A. Lomholt, and J.-H. Jeon, Bayesian inference of l\'evy walks via hidden markov models, arXiv preprint arXiv:2107.05390 (2021).
[10] H. Verdier, M. Duval, F. Laurent, A. Cassé, C. L. Vestergaard, and J.-B. Masson, Learning physical properties of anomalous random walks using graph neural networks, Journal of Physics A: Mathematical and Theoretical **54**, 234001 (2021).
[11] K. He, X. Zhang, S. Ren, and J. Sun, Deep residual learning for image recognition, in *Proceedings of the IEEE conference on computer vision and pattern recognition* (2016) pp. 770–778.
[12] T. Chen *et al.*, Guestrin, c.: Xgboost: A scalable tree boosting system, in *Proceedings of the 22nd ACM SIGKDD International Conference on Knowledge Discovery and Data Mining (KDD'16)* (2016) pp. 785–794.
[13] A. Argun, G. Volpe, and S. Bo, Classification, inference and segmentation of anomalous diffusion with recurrent neural networks, Journal of Physics A: Mathematical and Theoretical **54**, 294003 (2021).
[14] G. Muñoz-Gil, B. Requena, G. Volpe, M. A. Garcia-March, and C. Manzo, AnDiChallenge/ANDI_datasets: Challenge 2020 release (2020).
[15] M. Arts, I. Smal, M. W. Paul, C. Wyman, and E. Meijering, Particle mobility analysis using deep learning and the moment scaling spectrum, Scientific reports **9**, 1 (2019).
[16] I. Goodfellow, Y. Bengio, and A. Courville, *Deep Learning* (MIT Press, 2016) http://www.deeplearningbook.org.
[17] F. Chollet *et al.*, Keras, https://keras.io (2015).
[18] D. Li, Q. Yao, and Z. Huang, WaveNet-based deep neural networks for the characterization of anomalous diffusion (WADNet), Journal of Physics A: Mathematical and Theoretical 10.1088/1751-8121/ac219c (2021).
[19] J. Donahue, L. Anne Hendricks, S. Guadarrama, M. Rohrbach, S. Venugopalan, K. Saenko, and T. Darrell, Long-term recurrent convolutional networks for visual recognition and description, in *Proceedings of the IEEE conference on computer vision and pattern recognition* (2015) pp. 2625–2634.
[20] C. Manzo, Extreme learning machine for the characterization of anomalous diffusion from single trajectories (andi-ELM), Journal of Physics A: Mathematical and Theoretical 10.1088/1751-8121/ac13dd (2021).
[21] G.-B. Huang, Q.-Y. Zhu, and C.-K. Siew, Extreme learning machine: a new learning scheme of feedforward neural networks, in *2004 IEEE international joint conference on neural networks (IEEE Cat. No. 04CH37541)*, Vol. 2 (IEEE, 2004) pp. 985–990.
[22] G.-B. Huang, Q.-Y. Zhu, and C.-K. Siew, Extreme learning machine: theory and applications, Neurocomputing **70**, 489 (2006).
[23] S. Bai, J. Z. Kolter, and V. Koltun, An empirical evaluation of generic convolutional and recurrent networks for sequence modeling, arXiv preprint arXiv:1803.01271 (2018).
[24] N. Granik, L. E. Weiss, E. Nehme, M. Levin, M. Chein, E. Perlson, Y. Roichman, and Y. Shechtman, Single-particle diffusion characterization by deep learning, Biophysical Journal **117**, 185 (2019).
[25] E. Aghion, P. G. Meyer, V. Adlakha, H. Kantz, and K. E. Bassler, Moses, Noah and Joseph effects in lévy walks, New Journal of Physics **23** (2021).
[26] L. Chen, K. E. Bassler, J. L. McCauley, and G. H. Gunaratne, Anomalous scaling of stochastic processes and the moses effect, Physical Review E **95**, 042141 (2017).
[27] P. G. Meyer, V. Adlakha, H. Kantz, and K. E. Bassler, Anomalous diffusion and the Moses effect in an aging deterministic model, New Journal of Physics **20**, 113033 (2018).
[28] A. Gentili and G. Volpe, Characterization of anomalous diffusion classical statistics powered by deep learning (CONDOR), Journal of Physics A: Mathematical and Theoretical **54**, 314003 (2021).
[29] O. Garibo i Orts, M. A. Garcia-March, and J. A. Conejero, Efficient recurrent neural network methods for anomalously diffusing single-particle short and noisy trajectories, arXiv preprint arXiv:2108.02834 (2021).
[30] J. Lines, S. Taylor, and A. Bagnall, Time series classification with hive-cote: The hierarchical vote collective of transformation-based ensembles, ACM Trans. Knowl. Discov. Data **12**, 10.1145/3182382 (2018).
[31] T. Le Nguyen, S. Gsponer, I. Ilie, M. O'Reilly, and G. Ifrim, Interpretable time series classification using linear models and multi-resolution multi-domain symbolic representations, Data Mining and Knowledge Discovery **33**, 1183 (2019).

[32] P. Kowalek, H. Loch-Olszewska, and J. Szwabiński, Classification of diffusion modes in single-particle tracking data: Feature-based versus deep-learning approach, Physical Review E **100**, 032410 (2019).

[33] J. Janczura, P. Kowalek, H. Loch-Olszewska, J. Szwabiński, and A. Weron, Classification of particle trajectories in living cells: Machine learning versus statistical testing hypothesis for fractional anomalous diffusion, Physical Review E **102**, 032402 (2020).

[34] H. Loch-Olszewska and J. Szwabiński, Impact of feature choice on machine learning classification of fractional anomalous diffusion, Entropy **22**, 1436 (2020).
